# Exploring the Role of Anti-solvent Effects during Washing on Active Pharmaceutical Ingredient Purity

**DOI:** 10.1021/acs.oprd.1c00005

**Published:** 2021-03-12

**Authors:** Muhid Shahid, Georgia Sanxaridou, Sara Ottoboni, Leo Lue, Chris Price

**Affiliations:** † EPSRC Continuous Manufacturing & Advanced Crystallisation (CMAC) Future Manufacturing Research Hub, University of Strathclyde, Glasgow G1 1RD, U.K.; ‡ Department of Chemical and Process Engineering, University of Strathclyde, Glasgow G1 1XJ, U.K.

**Keywords:** isolation strategy, washing, anti-solvent effect, precipitation, optimization

## Abstract

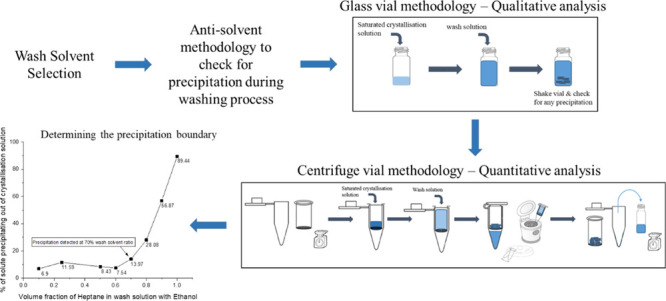

Washing is a key step in pharmaceutical isolation to remove the unwanted crystallization solvent (mother liquor) from the active pharmaceutical ingredient (API) filter cake. This study looks at strategies for optimal wash solvent selection, which minimizes the dissolution of API product crystals while preventing the precipitation of product or impurities. Selection of wash solvents to avoid both these phenomena can be challenging but is essential to maintain the yield, purity, and particle characteristics throughout the isolation process. An anti-solvent screening methodology has been developed to quantitatively evaluate the propensity for precipitation of APIs and their impurities of synthesis during washing. This is illustrated using paracetamol (PCM) and two typical impurities of synthesis during the washing process. The solubility of PCM in different binary wash solutions was measured to provide a basis for wash solvent selection. A map of wash solution composition boundaries for precipitation for the systems investigated was developed to depict where anti-solvent phenomena will take place. For some crystallization and wash solvent combinations investigated, as much as 90% of the dissolved PCM and over 10% of impurities present in the PCM saturated mother liquor were found to precipitate out. Such levels of uncontrolled crystallization during washing in a pharmaceutical isolation process can have a drastic effect on the final product purity. Precipitation of both the product and impurities from the mother liquor can be avoided by using a solvent in which the API has a solubility similar to that in the mother liquor; for example, the use of acetonitrile as a wash solvent does not result in precipitation of either the PCM API or its impurities. However, the high solubility of PCM in acetonitrile would result in noticeable dissolution of API during washing and would lead to agglomeration during the subsequent drying step. Contrarily, the use of *n*-heptane as a wash solvent for a PCM crystal slurry resulted in the highest amount of precipitation among the solvent pairs evaluated. This can be mitigated by designing a multi-stage washing strategy where wash solutions of differing wash solvent concentrations are used to minimize step changes in solubility when the mother liquor and the wash solvent come into contact.

## 1 Introduction

In the pharmaceutical industry, crystallization is a widely used purification technique employed to obtain active pharmaceutical ingredient particles of required size, purity, and crystal habit.^1,2^ Hence, crystallization has been extensively researched to establish an understanding and control of the key mechanisms that take place during this process to create the desired product with the requisite chemical and physical properties.^3^

Following crystallization, filtration, washing, and drying are the isolation steps required to separate the API crystals from the unwanted, impure mother liquor.^4^ Filtration uses a porous medium to retain the API crystals and separate them from the impure mother liquor which surrounds the crystals at the end of the crystallization process.^5^ Washing involves using a clean wash solvent to remove the unwanted impurities present within the mother liquors trapped between the API crystals in the filter cake. Drying is the final step required to remove the residual solvent (predominantly the wash solvent as most of the mother liquor will have been displaced during washing) from the API crystals forming the filter cake. The aim of drying is to produce a consistent, stable, and free-flowing product ready for secondary processing (formulation).^6^ Ideally, the complete isolation process should be achieved without any changes to the crystals produced during crystallization. Any breakage or granulation of crystals or precipitation of the dissolved product or impurities from the mother liquor onto the crystal surface should be avoided.^7^ Recently, attention has started to be paid to optimizing pharmaceutical isolation processes, especially filtration and drying. The major objective is to investigate the mechanisms affecting the product crystal attributes during these processes. This includes understanding the key mechanisms controlling the filtration and washing and involves designing continuous and semi-continuous filtration, washing, and drying rigs and investigating new analytical methods for effectively measuring the crystal product attributes obtained during and at the end of the isolation processes.^8−13^

Washing in pharmaceutical manufacturing is still relatively unexplored with very few academic publications.^14−16^ Washing plays a vital role in isolation since it is pivotal to the removal of impurities and the mother liquor from the API filter cake. The residual impure mother liquor present in the wet filter cake at the end of filtration contains any unreacted starting materials, unwanted side products (impurities of synthesis), and any degradants. If the remaining mother liquor is not removed, then the non-volatile dissolved materials would be deposited on the crystal surfaces during drying, increasing the impurity levels in the final isolated cake.^14^ This could result in the product failing to meet the purity requirements set out in the International Council for Harmonisation of Technical Requirement for Pharmaceuticals for Human use (ICH) Q6A guideline.^17^

Washing displaces the mother liquor present in the filtered cake with a wash solvent. This allows for the removal of dissolved raw material and impurities from the API crystal product. Tien (2012) proposed that washing of a filter cake is carried out by three main mechanisms: (1) displacement of mother liquor in the cake, (2) re-slurrying of the filter cake, and (3) consecutive dilution.^8,15^ During washing, the wash solvent first displaces the mother liquor from the large pores in the cake; then, the mother liquor from the adjacent narrower pores in the cake diffuses into the wash solvent. The resulting solute transport is regarded as axial dispersion. During subsequent washing steps, both diffusion and dispersion processes occur in combination.

To be effective, the wash solvent should ideally have the following properties:^18^

- Sufficient solubility of unwanted impurities to ensure they remain in the solution or dissolve;
- Low solubility of the API product to minimize product loss during the washing process;
- Miscibility with the mother liquor to allow diffusion and dilution mechanisms;
- The viscosity of the wash solvent should be similar to that of the crystallization solvent to allow for appropriately long contact with the crystals to allow the removal of impurity from the cake without excessive filtration cycle time;^14^
- The API product should have thermal stability in the wash solvent under drying process conditions needed to remove the wash solvents;
- The volatility of the wash solvent should be kept appropriately low to assist with the drying process.

However, some of these wash characteristics are mutually exclusive. Introduction of the wash solvent into the mother liquor wet API filter cake can result in several undesirable outcomes. The anti-solvent effect is one of the problems commonly encountered during washing because of the requirement for the API product to have a low solubility in the wash solvent. As the wash solvent comes in contact with the slightly supersaturated mother liquor present within the filter cake, nucleation takes place initiating anti-solvent crystallization. Anti-solvent crystallization, also known as precipitation, is a widely used technique in the pharmaceutical and fine chemical industry to recover a product from solution in a solvent in which the product has high solubility.^19^ Supersaturation is generated by mixing a concentrated solution of the product with another miscible solvent in which the product has limited solubility. Anti-solvent crystallization can be well controlled and avoids the need to heat and cool the product stream where this is undesirable.^20^ However, this control of anti-solvent crystallization is lacking during washing and is made more difficult in binary solvent mixture systems due to a non-linear relationship between solubility and composition. Rather, washing with an anti-solvent may lead to uncontrolled anti-solvent crystallization and can result in product precipitation, leading to severe agglomeration.^18^ Precipitation occurring in the packed bed of API crystals in the filter cake provides ideal conditions for the formation of solid bridges between crystals and hence agglomerate formation during washing. With a high impurity content present in the mother liquor, the function of the wash as an anti-solvent can have a drastic effect on the purity of the final product as the impurities are potentially subject to anti-solvent crystallization.

One solution to this problem is to use a chilled crystallization solvent as the first wash solvent, possibly saturating the crystallization solvent to minimize the extent of dissolution. An alternative is selecting a solvent in which the API has similar solubility as that of the API in the mother liquor to prevent precipitation of material present within the mother liquor solution.^9^ However, these approaches result in a reduction of yield from that obtained during the crystallization process. In addition, the final crystalline product obtained at the end of the drying process could have a different particle size distribution to the one obtained during crystallization.

**Figure 1:**
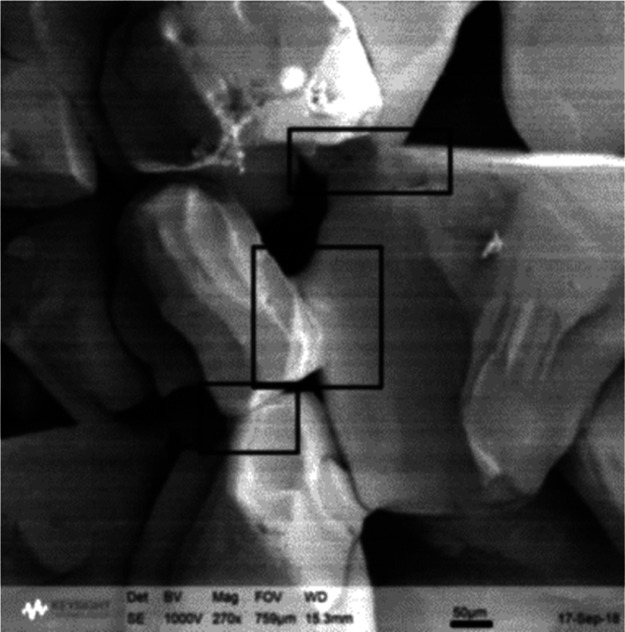
SEM image of the dried PCM agglomerate showing the formation of crystal bridges.

A confounding factor in investigating the anti-solvent effect during washing leading to agglomerate formation is that agglomerates can also be formed during crystallization and carried into the isolation process and, furthermore, agglomerates can be formed during drying. The presence of dissolved API in the residual wash solvent in the washed cake at the start of the drying process results in agglomeration as crystalline bridges form as the wash solvent evaporates from the porous crystal structure.^21,22^ The capillary forces acting on the retained liquid film in the wet cake tend to concentrate the residual solution at the points of contact between particles favoring agglomerate formation. This effect is illustrated in the SEM image shown in Figure 1. This illustrates the presence of crystalline bridges in the API paracetamol (PCM) at the end of drying (PCM API present in ethanol as the crystallization solvent and washed using acetonitrile and dried in a vacuum oven). The presence of crystalline bridge formations produced during any of the process steps, crystallization, washing, and drying, will increase the particle size of API crystals, which are typically characterized at the end of the drying process.^10^ To overcome the consequences of agglomeration, milling is often used as a downstream process step in the pharmaceutical industry applied either after drying or immediately prior to formulation. This not only increases the number of process steps and processing time but can also lead to the formation of amorphous materials and expose new facets of the crystals, which may have different characteristics to those formed during the crystallization and may modify the powder behaviors.^23^

This suggests that poorly designed washing processes can modify the particle properties. This is a potentially important complication in the subsequent drying step, which is very likely to further strengthen the agglomerates formed during washing. Therefore, it is important to prevent agglomeration during washing and limit the amount of product in solution at the start of drying by optimizing the washing process. This research investigates strategies to follow for optimal selection of the wash solvent. The approach involves minimizing the dissolution of API crystals while preventing any precipitation of dissolved API and impurities. Selecting a wash solvent to avoid these phenomena when the wash solvent comes into contact with the retained crystallization solvent in the saturated filtered cake can be challenging but is essential to maintain the yield, purity, and particle characteristics throughout the isolation process.

This study investigates the effect of wash solvent selection by considering the mechanisms taking place during the interaction between crystallization and wash solvent in the washing process. In the first instance, the already crystallized API particles in the filter cake present during the washing process are ignored to simplify the system. Once the processes taking place in the liquid solvent mixture, during the washing process, are understood, then the API crystals in the cake can be reintroduced and the understanding of the solution side processes can be built upon.

The aim of this work is to develop a quick and simple screening methodology to qualitatively and quantitatively analyze the propensity of different wash solvents to cause precipitation to occur during the washing process. PCM was the API selected for the experimental work in this study as it is a widely researched compound with a significant body of published data, which can be drawn upon to facilitate the experimental work.^24−27^

The approach developed in this study allows quantification of both the amount of PCM API precipitating out during washing and the quantity of dissolved impurities that could precipitate out and adversely affect the purity of the final product. The findings from this work allow crystallization–wash solvent combinations which would prevent/limit any precipitation or dissolution during washing to be identified. This is illustrated with PCM as a model compound.

## 2 Materials and Methods

### 2.1 Raw Material

PCM was selected as a representative test compound with characteristics typical of APIs. It is commercially available, as are its impurities of synthesis. In this study, PCM of typical crystalline grade was used (Mallinckrodt Inc., UK, batch 637514D001; *x*_10_: 12.48 μm, *x*_50_: 43.96 μm, *x*_90_: 101.30 μm). Acetanilide (Sigma-Aldrich, UK, lot # STBF7835V, purity 99%) and metacetamol (Sigma-Aldrich, UK, lot # MKBX4643V, purity 97%), two structurally related compounds to PCM, were used as representative impurities in this work. These impurities could be present in the mother liquor during the crystallization step.^28^

To investigate the anti-solvent effect during washing on representative slurry suspensions typical of those formed at the end of crystallization, a series of three commonly used crystallization solvents appropriate for isolating PCM were used as follows: ethanol (purity ≥ 99.8% (GC), from Sigma-Aldrich), propan-2-ol (IPA) (purity ≥ 99.5% (GC), from Sigma-Aldrich), and 3-methylbutan-1-ol, (isoamyl alcohol) (purity ≥ 99.5% (GC), from Sigma-Aldrich).^29^ As wash solvents, acetonitrile (purity 99+ % from Alfa Aesar), isopropyl acetate (purity 99+ % from Alfa Aesar), and *n*-heptane (purity 99%, from Alfa Aesar) were selected for this investigation. Acetonitrile was chosen because the API solubility is at the high end of those typically selected as wash solvents and because it is a widely used solvent in industry. *n*-Heptane was selected because the solubility of PCM and selected impurities is very low, almost negligible. Isopropyl acetate is another commonly used wash solvent in industry and the solubility of API in the isopropyl acetate is in the middle of the two extremes represented by acetonitrile and heptane. A further criterion is that all three wash solvents were chosen to be miscible with the three crystallization solvents.

To determine the purity of the precipitated material at the end of each experiment, high-pressure liquid chromatography (HPLC) was used. The eluents contained water (water, ultrapure, HPLC grade, Alfa Aesar) and methanol (methanol, ultrapure, HPLC grade, 99.8+%, Alfa Aesar). Methanol was also used as a diluent for some samples.

### 2.2 Sample Preparation

A saturated PCM solution, with impurities included where selected, was prepared in two stages based on previously measured solubility of PCM in the selected crystallization solvents:^30^ First, 2% by mass relative to the known PCM solubility of each impurity was added and dissolved in the crystallization solvent. To ensure complete dissolution of impurities, a sonic water bath was used (Elmasonic P300H Ultrasonic, Cole-Parmer Instruments Ltd.). The amount of PCM required to saturate the solution was then added and dissolved in a similar manner. This two-stage addition prevents any undissolved impurity crystals remaining in the final saturated solution. The saturated solution was also filtered before anti-solvent screening experiments to prevent the potential seeding effect.

Wash solutions were prepared using a mixture of the selected wash solvent and crystallization solvent. For each crystallization and wash solvent system, a total of eight different wash solution combinations were investigated. Different ratios, by volume, of wash solution used in each solvent system are reported in Table 1.

**Table 1:** Wash Solutions of Different Ratios That Were Tested for Each Solvent System

**wash solvent solution identity**	**percentage of crystallization solvent by volume (%)**	**percentage of wash solvent by volume (%)**
1	90	10
2	75	25
3	50	50
4	40	60
5	30	70
6	20	80
7	10	90
8	0	100

### 2.3 Anti-solvent Screening Procedure

Two anti-solvent screening approaches were developed and evaluated, one based on portion-wise addition of the wash solvent to a saturated solution and monitoring by visual observation and the other used centrifugation to separate and recover any precipitated particles.

#### 2.3.1 Anti-solvent Screening Procedure—Glass Vial Method

**Figure 2:**
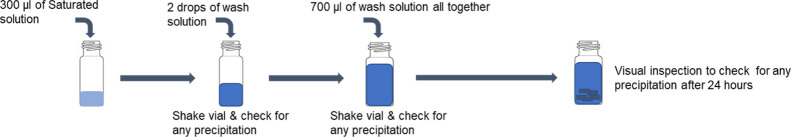
Glass vial precipitation detection method.

Figure 2 gives a schematic representation of the glass vial method. This method uses a standard 1.8 mL glass HPLC vial. 300 μL of saturated crystallization solution is first added to the vial using an Eppendorf pipette. Then, the wash solution is added 2 drops at a time using a 1 mL disposable pipette. After each addition, the vial was shaken and checked for any precipitation of crystals that might have taken place. Wash solvent addition was continued until the total amount of wash solvent added corresponded to 700 μL. Given that a typical saturated filter cake contains very approximately 50% by volume API crystals and 50% by volume mother liquor, then a one cake volume wash would broadly match the 2:1 ratio achieved here depending on particle aspect ratio and packing. The amount of wash solvent used is better expressed as a two-cake void volume wash.^31^ The glass vial was then visually inspected at the end of the drop-wise addition to check for any precipitation of crystals. If no crystals were formed, the vials were re-inspected the following day (approximately 24 h later) to determine whether precipitation was possible but it was a very slow process under the conditions investigated. While precipitation taking significantly longer than the normal duration of the washing step may not be of practical significance, it is considered to be useful to know whether precipitation is possible under each of the conditions investigated.

#### 2.3.2 Anti-solvent Screening Procedure—Centrifuge Vial Method

To evaluate the anti-solvent effect during washing using a centrifuge tube setup, centrifuge filter tubes incorporating a basket with a 0.2 μm pore size were used (Thermo Scientific National, Scientific F2517-9 X100 PTFE 750 μL Centri Filter 0.2 μm pore size). The small pore size allowed for mixing of the sample solution and the wash solvents to be performed in the filter basket without any solvent leakage into the filter tube.

**Figure 3:**
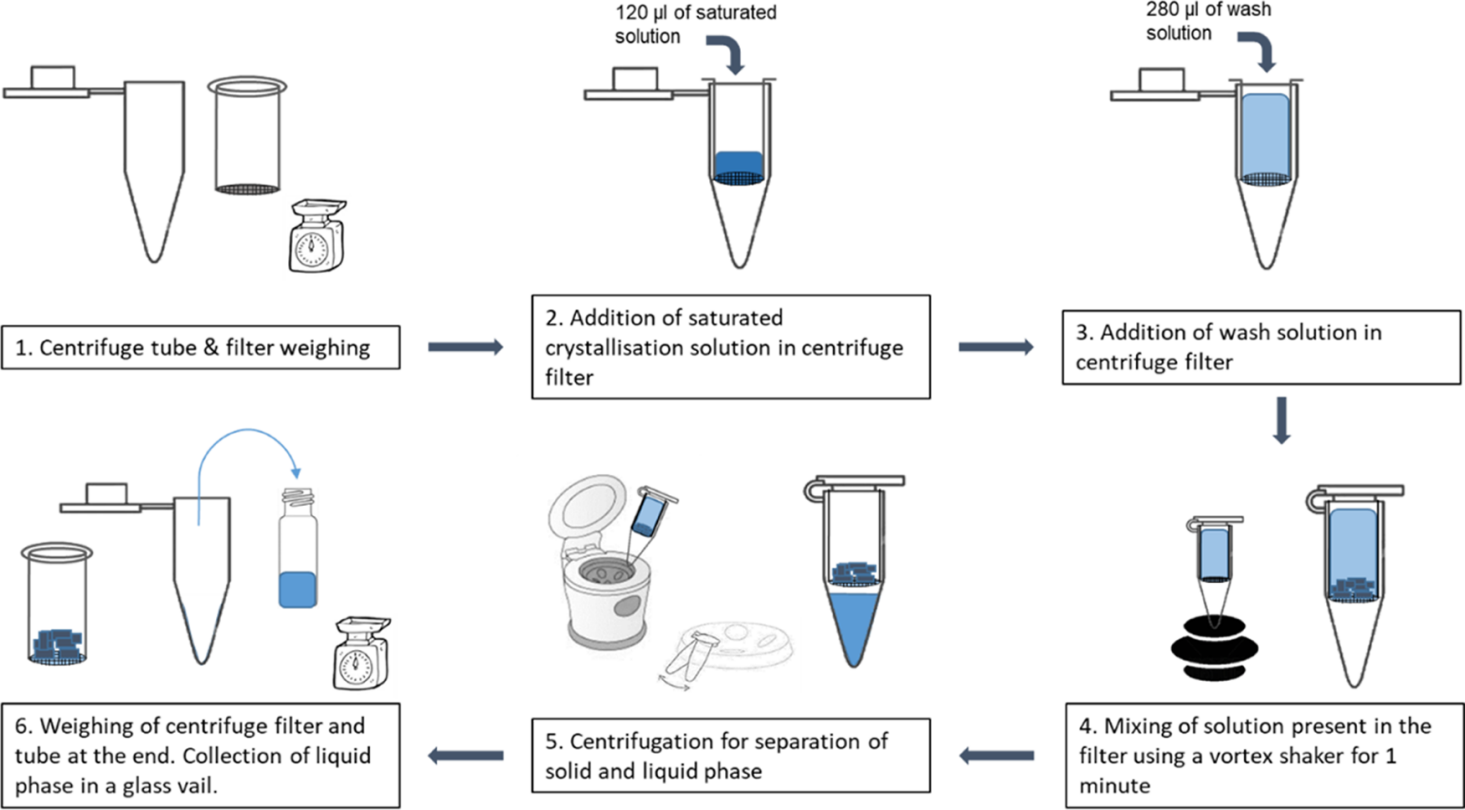
Centrifuge vial precipitation detection method.

Figure 3 is a schematic representation of the anti-solvent methodology developed using the centrifuge vial. The procedure was divided into six steps, with a mass balance maintained across each step to take into account any material loss. In a pre-weighed centrifuge filter basket and centrifuge tube, the saturated crystallization solvent was added and the mass of the filled tube was recorded (Figure 3).

The centrifuge filter basket had a capacity of 500 μL; thus 120 μL of saturated crystallization solvent was added using an Eppendorf pipette; this was followed by the addition of 280 μL of the wash solvent. The choice of solvent volumes allowed a small space to remain at the top of the filter basket to prevent any solvent spillage while mixing the sample using a vortex shaker.

After addition of the wash solution (step 4 in Figure 3), the solvent was kept in the centrifuge tube basket for 2 h and then the anti-solvent effect was checked (looking for any crystal formation). Longer contact times between the mother liquor and the wash solvent, for example 24 h, were not investigated as the selected centrifuge vials were found not to seal well enough to completely prevent solvent evaporation occurring if vials were left overnight. Also, the filter medium in the baskets eventually allowed the solvent to drain onto the centrifuge vial due to gravity if left over a long period of time. The compromise of 2 h was selected as an appropriate amount of time to represent the practical maximum time for which the wash solvent would be present in contact with the saturated crystallization solution in the API cake.

The separation of any precipitated solid from the mixture of the saturated solution and the wash solvent takes place in step 5 of Figure 3. Centrifugation was carried out for 2 min at 6000 rpm. The basis of selection of these conditions is reported in the Supporting Information. The chosen conditions were found to be effective in separating the mixed crystallization and wash solvent from any precipitated solid particles retained in the centrifuge filter basket.

### 2.4 Post-anti-solvent Procedure Analysis

HPLC was used to investigate the composition of the liquid and solid phases obtained using the centrifuge vial method. X-ray powder diffraction (XRPD) and differential scanning calorimetry (DSC) were performed on the precipitated solid phase to determine the crystal structure and to investigate whether the impurities present in the saturated solution were present as separate crystals or incorporated within the PCM API crystal lattice.

HPLC was used to determine the concentration of PCM and its impurities present in the liquid and solid phases at the end of the anti-solvent screening methods. Water and methanol were used as the eluents in the mobile phase, whereas methanol was also used as a diluent for the samples. Calibration curves for pure PCM, metacetamol, and acetanilide were gathered using a multilevel calibration method reported in the Supporting Information. An Agilent 1260 Infinity II system was used. The column was an Agilent Poroshell 120 EC-C18 4.6 × 100 mm 4 μm operated at 40 °C with a flow rate of 1 mL/min. The injection volume was 5 μL; data were collected at 243 nm wavelength; and the mobile phase was 80% water and 20% methanol.

XRPD analysis was performed using a D8 (multi-well) powder X-ray diffractometer—flat plate instrument, Bruker AXS GmbH. The detector rotation (2θ) was set to 2θ_min_ at 4^°^ and 2θ_max_ to 35^°^. A step size of 0.017^°^ was used and the sec/step was set to 1 s.

DSC analysis was performed using a DSC 214 Polyma, NETZSCH–Gerätebau GmbH. Standard aluminum pans were used. The mass of the sample added to the pans was maintained at around 2–3 mg. The DSC214 Polyma employed a helium purge (inline pressure set to 0.5 bar) and a protective gas during analysis, flowing through a chiller unit for sample cooling. The initial temperature was set to ambient, 25 °C, and the final temperature was set to 200 °C. The heating rate used was 10 °C/min. A sample was also run with a heating rate of 2 °C/min to check the sensitivity, looking for peak separation that might be missed at a high heating rate.

### 2.5 Gravimetric Solubility Analysis Procedure

The solubility of PCM in the binary solvent mixtures (crystallization and wash solvents) was determined experimentally by equilibration and gravimetric analysis. A Hailea HC-100A chiller was used to maintain the temperature at 22 °C (the average temperature of the lab where the anti-solvent screening experiments were conducted). Excess PCM was added to 20 mL, clear glass vials together with the binary solvent mixture and a magnetic stirrer bar. The vials were sealed and left on a multi-position stirrer plate inside the water bath for around 48 h to equilibrate. Samples of the solutions were then taken from the slurry in the vials using a syringe, filtered using a PES syringe filter (Fisherbrand, cat no. 15206869, 0.2 μm, sterile), and added to a separate glass vial which was weighed and then left to dry. Table 1 shows all the ratios of binary solvent mixtures for which solubility was determined.

## 3 Results and Discussion

### 3.1 Anti-solvent Effect—Glass Vial Method

**Figure 4:**
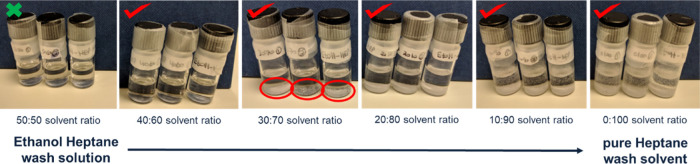
Ethanol–*n*-heptane glass vial precipitation qualitative test.

Figure 4 shows the results from the anti-solvent screening carried out for the ethanol–*n*-heptane solvent system using the glass vial method. The 50:50 solvent ratio (first picture on the left in Figure 4) represents a wash solution made up of 50% by volume ethanol (the crystallization solvent) with 50% by volume of *n*-heptane (the wash solvent), respectively. Precipitation was first observed in the ethanol–*n*-heptane experiments when a wash solution ratio of 40:60 was used (40% ethanol and 60% *n*-heptane by volume). In this condition of 40:60 wash solution, local and rapid precipitation of crystals was observed as the first few drops of the wash solution were added to the saturated crystallization solvent. These crystals subsequently dissolved back into the mixed liquid phase after a few seconds, once all the wash solution was added to the saturated crystallization solvent. Therefore, the initial precipitation observed was due to the local supersaturation in a non-mixed environment. As soon as mixing occurred, the bulk composition remained undersaturated; consequently, the crystals dissolved back in the solution. However, after leaving the vials for 24 h, three or four small crystals were seen at the bottom of the glass vial by the naked eye. This delayed precipitation indicates the slow kinetics of the system at this composition.

For the samples with compositions of 30:70 to 0:100 ethanol/*n*-heptane in Figure 4, crystal precipitation occurred as soon as the wash solution was added and mixed with the saturated crystallization solvent. This was due to the large solubility difference between the crystallization solvent and the wash solutions. The crystals formed can be seen at the bottom of the vials as indicated by the red circle in the 30:70 solvent ratio vials in Figure 4.

There is an increase in crystal concentration in the vials going from compositions of 40:60 to 0:100 ethanol/*n*-heptane as seen in Figure 4. This increase is due to the higher supersaturation achieved in the solvent mixture as the concentration of *n*-heptane in the wash solution increases, which can be seen in Table 4. Higher supersaturation results in a more thermodynamically unstable solution, which then results in increased precipitation of crystals occurring to allow the solution to return to thermodynamic equilibrium.^32^

This anti-solvent effect (crystal formation due to anti-solvent addition) was seen for five different cases for the combination of crystallization solvent and wash solvents used in this study. The results for this can be seen in Table 2. For each solvent combination case, if precipitation of crystals is observed, then the solvent composition or the solvent proportions of the wash solution at the point where precipitation is first observed is given in Table 2.

**Table 2:** Precipitation Caused for Different Solvent Combinations—Glass Vial Method^a^

		*wash solvent*		
		*n-heptane*	*acetonitrile*	*isopropyl acetate*
**crystallization solvent**	**ethanol**	**40**–*60%* (v/v)	no nucleation	**10**–*90%* (v/v)
	**isopropanol**	**40**–*60%* (v/v)	no nucleation	**0**–*100%* (v/v)
	**isoamyl alcohol**	**20**–*80%* (v/v)	no nucleation	no nucleation

a The crystallization solvent used is provided on the left side of the table, while the wash solvent is given across the top of the table. The ratio of the wash solution at which precipitation is first observed in the solvent system for the PCM API case is given here. (The bold numbers correspond to the volume ratio of the crystallization solvent in the wash solution, while the italic number corresponds to the volume ratio of the wash solvent in the wash solution.)

In the case of using *n*-heptane as the wash solvent, precipitation was detected in all three different crystallization solvent systems. The almost negligible solubility of PCM in *n*-heptane combined with its much higher solubility in the crystallization solvents results in a supersaturated solution being formed as the wash solution is added to the saturated crystallization solvent. Table 3 provides the experimentally determined solubility of PCM in all six pure solvents used in this study. Table 4 provides the change in saturation (Δ*C*) in the final solution obtained at the end of washing after all the wash solution is added for all the different wash ratios used.

**Table 3:** Experimental Solubility Determined for PCM in Selected Solvents at 22 ^°^C (Average Lab Temperature at Which This Anti-solvent Effect Study Is Conducted)

	**solvent**	**solubility (g API/g solvent) (at 22 °C)**
**crystallization solvent**	ethanol	0.1867
	isopropanol	0.1141
	isoamyl alcohol	0.0526
**wash solvent**	acetonitrile	0.0240
	isopropyl acetate	0.0059
	*n*-heptane	0.0003

**Table 4:**
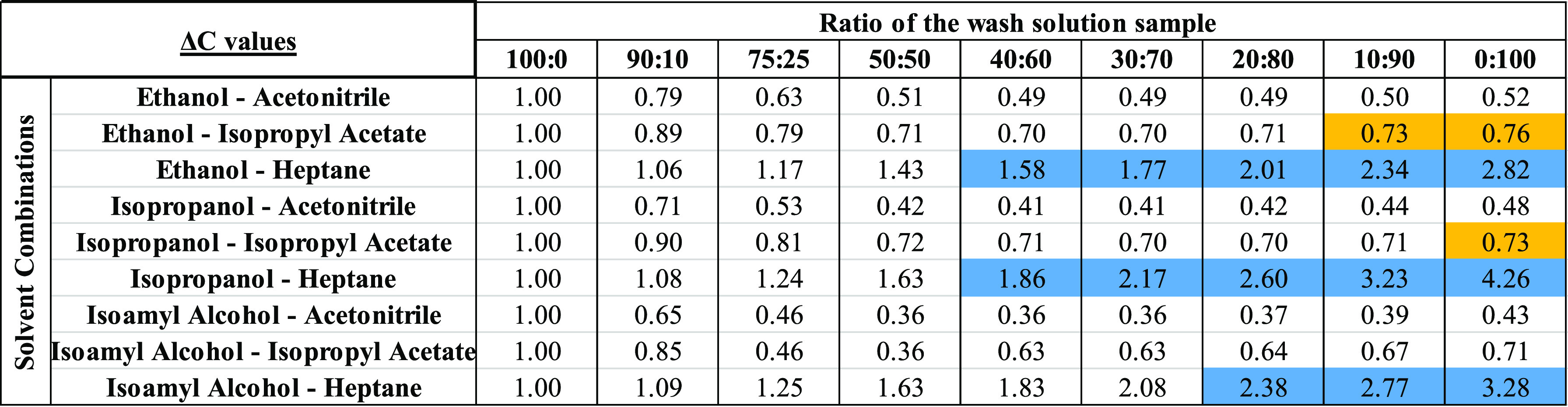
Δ*C* Achieved for the Solvent Combinations Used^a^

a Blue cells represent scenarios where nucleation and crystallization were observed. Orange cells represent scenarios where local supersaturation resulted in nucleation and then dissolution of crystals as bulk saturation is reached.

There was no anti-solvent effect observed where acetonitrile was used as the wash solvent. Acetonitrile has the highest solubility of all the wash solvents used. Looking at the binary solvent solubility graphs for the acetonitrile cases (see the Supporting Information), the operating dilution line is below the solubility curve. This explains why precipitation cannot take place as the concertation of PCM in this system remains below the solubility limit of the solution. The calculated Δ*C* values for all acetonitrile cases, Table 4, shows that supersaturation is not achieved and so no precipitation should be observed. In fact, any PCM crystals present would be subject to dissolution in these unsaturated conditions.

As the calculated Δ*C* values (Table 4) for all cases with isopropyl acetate as the wash solvent was also found to be <1 for all the wash solution ratios, no precipitation should have been detected. However, during the glass vial experiment, for both ethanol and isopropanol crystallization solvent cases with isopropyl acetate, a few crystals were seen to form as the first few drops of wash solution was added to the saturated solvents, as reported in Table 2. This phenomenon probably occurred due to the local supersaturation effect where the localized environment close to the site of the drop addition would have resulted in nucleation of crystals due to poor mixing. This then seems to disappear after the whole wash solution was added and the solution in the vial had become fully mixed. This effect if encountered during washing an API filter cake, where solution mixing can be very limited, could have a detrimental effect on the purity and particle size distribution of the final isolated product.

The glass vial method used for anti-solvent effect screening was found to be effective for qualitative analysis of the wash solvent effect. The precipitation of crystals formed due to interaction between the wash solution and the mother liquor is observable and this method can be used as a “quick” first approach to assess wash solvent compatibility.

However, quantitative analysis to determine the amount and identity of solute precipitating out of the solution required a different methodology. The complete separation of the solid from the liquid solution in the glass vial is not a trivial procedure due to the small size of the vial and due to the difficulties related to the separation of the liquid and solid parts of the sample. This methodology, therefore, does not allow a precise quantification of the species precipitated because the data generated using HPLC gave inconclusive evidence on the amount of impurities precipitating out, as the solid analysis results were affected by residual liquid solvent still present at the bottom of the vials (see the Supporting Information). To get a better quantitative result, an improved wash screening analysis was devised to overcome these separation issues. Hence, the centrifuge vial method was developed.

Table 5 shows the anti-solvent effect observed using the centrifuge vial method. This is similar to the glass vial method described in Table 2. Due to the opaque character of the polypropylene centrifuge vials, nucleation and crystallization phenomena were much harder to observe compared to using the clear glass vials.

**Table 5:** Precipitation Caused by Different Solvent Combinations—Centrifuge Vial Method

		*wash solvent*		
		*n-heptane*	*acetonitrile*	*isopropyl acetate*
**crystallization solvent**	**ethanol**	**30**–*70%* (v/v)	no nucleation	no nucleation
	**isopropanol**	**30**–*70%* (v/v)	no nucleation	no nucleation
	**isoamyl alcohol**	**10**–*90%* (v/v)	no nucleation	no nucleation

a The crystallization solvent used is reported on the left side of the table, while the wash solvent is given across the top of the table. The ratio of the wash solution at which precipitation is first observed in these solvent systems for PCM as a representative API is reported here. (The bold numbers correspond to the volume ratio of crystallization solvent in the wash solution, while the italic number corresponds to the volume ratio of wash solvent in the wash solution.)

### 3.2 Anti-solvent Effect—Centrifuge Vial Method

Comparing the results shown in Table 5 with those obtained using the glass vial method, (see Table 2), reveals some differences. Due to the opaque nature of the centrifuge vials, the nucleation observed due to local supersaturation effects for isopropyl acetate, which was seen in the glass vial method, could not be discerned in the centrifuge vial experiment. In all the cases of *n*-heptane as the wash solvent, nucleation was observed for wash solution ratios with a higher *n*-heptane concentration. This offset in observation of the anti-solvent effect can again be attributed to the opaque nature of the centrifuge vials giving difficulties in visualization of precipitation of few, small crystals. Also, the slow crystallization kinetics noticed in the 40:60 ethanol/*n*-heptane case in the glass vial experiments is not noticed in the centrifuge method as the solution is only left for 2 h compared to 24 h in the glass vial method.

However, the quantitative analysis achieved using the centrifuge vial method was found to be much more successful as almost complete separation of solid crystals from liquid solution was achieved. After stage 6 in Figure 2, HPLC is performed on both the separated solid and liquid samples. Quantitative results obtained from two different scenarios, ethanol–acetonitrile and ethanol–*n*-heptane, are given below. The two scenarios presented illustrate the results which would be obtained for most cases depending on whether precipitation is observed or not. The results for all the samples and for all solvent combinations are available in the Supporting Information.

#### 3.2.1 Centrifuge Vial Method—Quantitative Analysis: Ethanol–Acetonitrile (No Nucleation)

**Figure 5:**
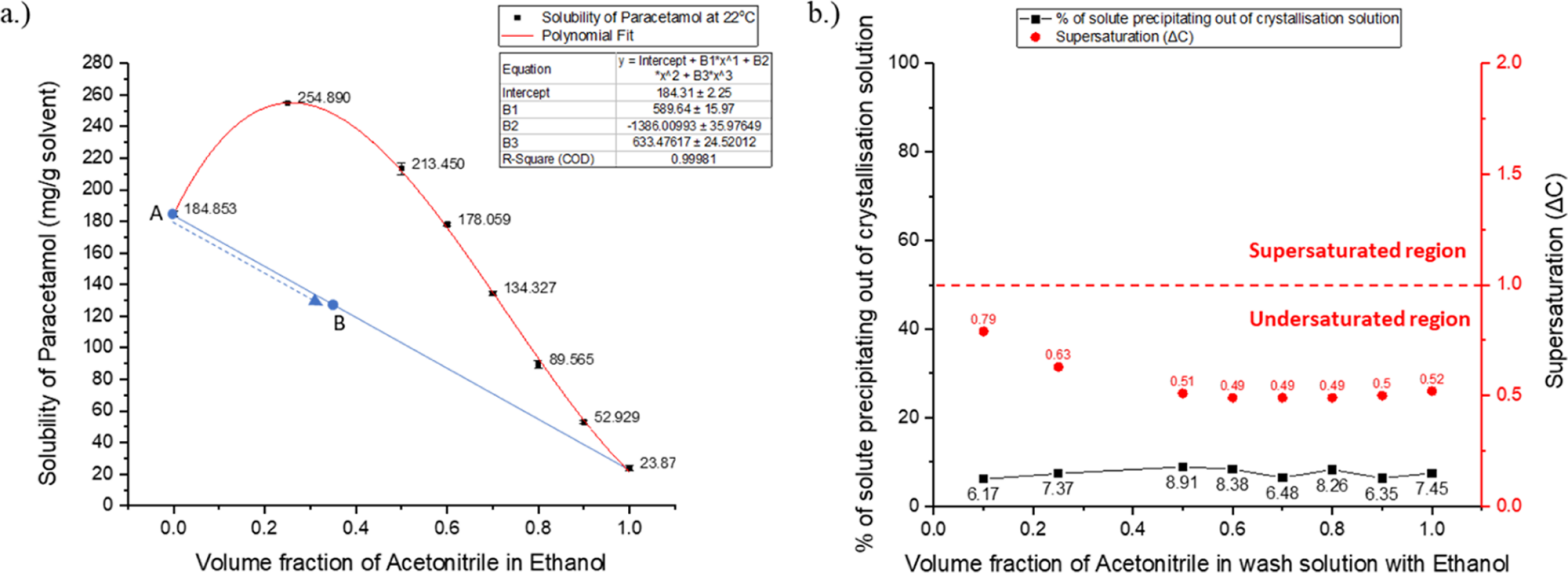
Quantitative analysis of the ethanol–acetonitrile case. (a) Solubility of PCM in an ethanol–acetonitrile binary solvent mixture at 22 °C. (b) Percentage of solute precipitating out of solution for different wash solution compositions is shown in the *Y* axis on the left-hand side of the graph with the supersaturation achieved in the solution when different ratios of wash solution are added to the saturated crystallization solvent shown on the *Y* axis on the right-hand side of the graph.

As reported in the binary solvent mixture solubility data reported in Table 6 and in Figure 5, the ethanol and acetonitrile system does not show any anti-solvent effect. The blue line in Figure 5, graph a, represents the change in concentration of the API in the resultant solution mixture as the wash solution is added to the saturated crystallization solvent. Point A in the graph is the starting API concentration of the saturated ethanoic solution. As the wash solution is added to the saturated crystallization solvent, the concentration of API in the solution will change and move down following the path of the blue line. The calculated end point of the overall solution is dependent on the wash solution that is used, given in Table 6. Hence, the end point of the API concentration in the new system (mother liquor and wash solvent) depends on the composition and quantity of the wash solution being used (in this study, the total volume ratio of the system is fixed at 700 μL of wash solution to 300 μL of mother liquor). For example, if an experiment with the wash solution comprising equal volumes of the wash solvent and the crystallization solvent is used, then looking at Table 6, the final composition of the overall solution (containing the saturated crystallization solution and the wash solution) would be 35% by volume acetonitrile in ethanol. The value of PCM concentration at 35% by volume wash solvent can then be determined from the blue line in graph a of Figure 5, which would correspond to around 127 mg of PCM/g solvent, point B.

**Table 6:** Ratio of Wash Solvent in the Final Solution Mixture

**ratio of wash solution used** (v/v) (crystallization/wash)	90:10	75:25	50:50	40:60	30:70	20:80	10:90	0:100
**volume fraction of wash solvent in final solution (end point of the final solution)**	0.07	0.175	0.35	0.42	0.49	0.56	0.63	0.7

The difference between the diluting line and the solubility curve dictates whether precipitation could take place. For the case of ethanol and acetonitrile (Figure 5), since the diluting line is below the solubility curve, the actual concentration of PCM in system is below the solubility limit. Therefore, the solution would be undersaturated and no precipitation would occur.

In Figure 5b, the red dots represent the corresponding supersaturation across the solvent composition investigated, showing no precipitation of the API or the impurity as the system is in the undersaturated region. Table 7 shows the solubility data of the API and the selected impurities in the pure solvents used in this study. Since the solubility of the impurities, metacetamol and acetanilide, in the pure solvent is similar to or greater than that of PCM and only 2% by mass of impurity is present in each crystallization solution, any impurity present in the precipitated material would be due to incorporation in API crystals rather than independent crystallization of the impurities as separate crystalline species.

**Table 7:** Experimental Solubility Determined for Metacetamol and Acetanilide in Selected Solvents at 25 °C

	solvent	solubility (gPCM/g solvent) (at 25 °C)	solubility (g metacetamol/g solvent) (at 25 °C)	solubility (g acetanilide/g solvent) (at 25 °C)
**crystallization solvent**	ethanol	0.2057	0.2944	0.3322
	isopropanol	0.1243	0.1948	0.1957
	isoamyl alcohol	0.0549	0.1049	0.1656
**wash solvent**	acetonitrile	0.0294	0.0776	0.2060
	isopropyl acetate	0.0076	0.0246	0.0896
	*n*-heptane	0.0003	0.0003	0.0004

Even though there is no precipitation observed in the ethanol–acetonitrile case, the measured percentage precipitation value remains constant at around 7 ± 1% as indicated by the black squares in graph b of Figure 5. This consistent amount of precipitation along the varying wash solution compositions used can be explained by the presence of the crystallized material formed from the solution left on the porous media of the centrifuge vial basket. This crystallization is therefore occurring during the solvent evaporation.

Since no precipitation takes place in the ethanol–acetonitrile solvent combination, this does not automatically make acetonitrile a good candidate as the wash solvent for PCM in the ethanol crystallization solvent. Selecting a wash solvent with a moderate solubility of PCM API can reduce the isolation yield by dissolution of the particles forming the API cake. Figure 5, graph a, shows that the operating dilution line is below the solubility curve and so the acetonitrile wash solution would tend to dissolve some of the PCM crystals present in the filter cake. Also, and probably of greater importance, the residual acetonitrile wash solution left in the deliquored cake would likely result in particle agglomeration during the drying process. Evaporation of the residual wash solution in the API cake would cause crystallization of the dissolved solute on the crystal surfaces forming crystal bridges in the API cake (as seen in Figure 1).

#### 3.2.2 Centrifuge Vial Method—Quantitative Analysis: Ethanol–*n*-Heptane

**Figure 6:**
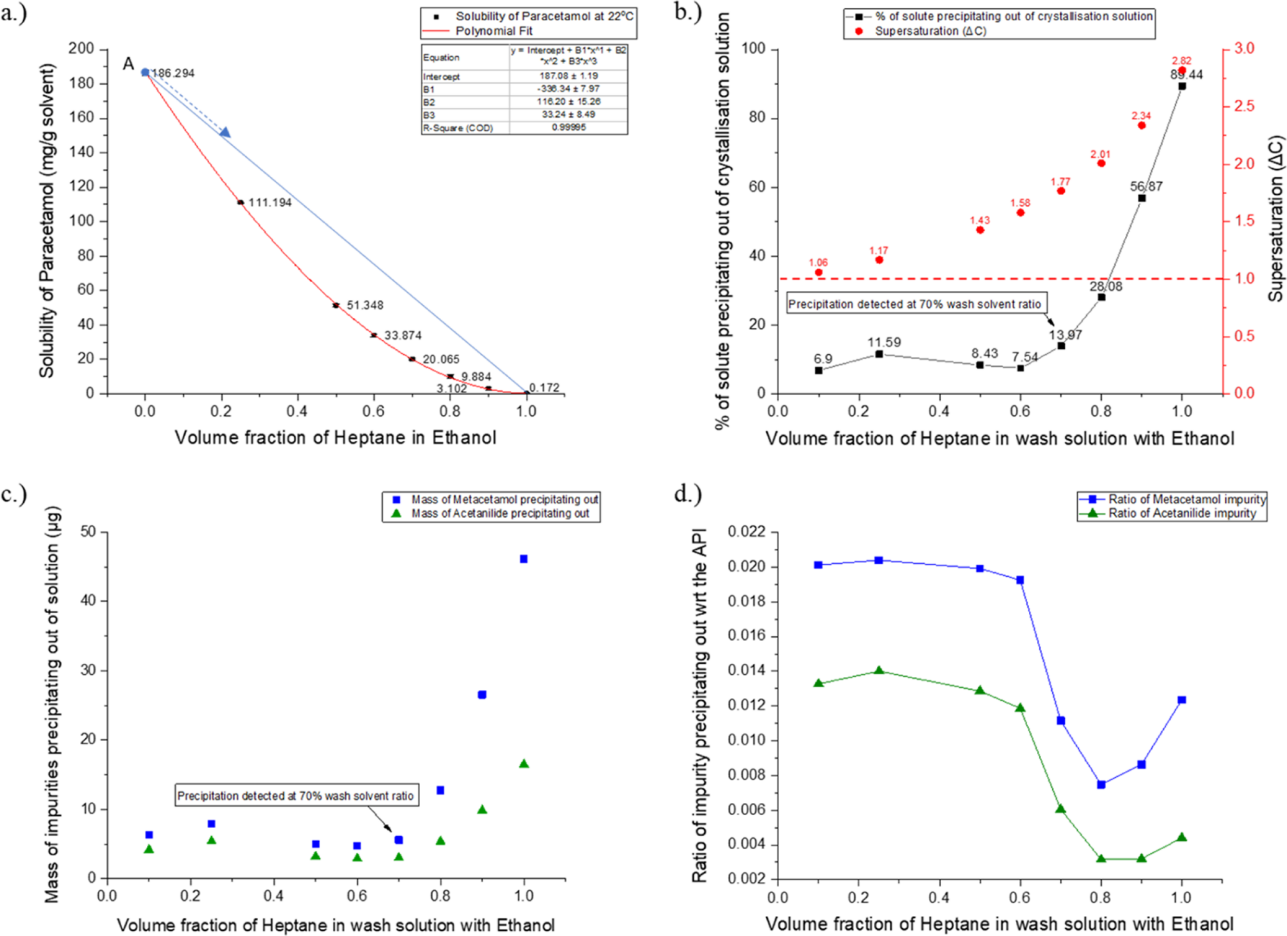
Quantitative analysis of the ethanol–*n*-heptane case. (a) Solubility of PCM in the ethanol–*n*-heptane binary solvent mixture at 22 °C. (b) Percentage of solute precipitating out of the solution for different wash solution compositions is shown in the graph together with the supersaturation achieved in the solution when different ratios of wash solution are added to the saturated crystallization solvent. (c) Mass of impurities precipitating out when using different ratios of wash solution. (d) Ratio of impurities precipitating out with respect to the PCM (API) precipitating out for each of the different ratios of wash solutions used.

As reported in Figure 4 and validated by the PCM solubility data determined for the ethanol–*n*-heptane binary solvent mixture (Figure 6, graph a), precipitation was detected. The figure shows PCM supersaturation was generated as the wash solution is added to the saturated crystallization solution. The end point composition of the solution on the operating dilution line would be dependent on the ratio of wash solution added (Table 6). Since the API concentration in the system would be higher than the solubility of the API in the solution (blue dilution line above the solubility curve), supersaturation would be generated and precipitation would likely be observed.

Graph b in Figure 6 shows the percentage of solute precipitating out of the solution (black line with square points) and the supersaturation level reached (red dots) for the different ratios of wash solution used. Precipitation of the solute was detected after a wash solution of 60% *n*-heptane and 40% ethanol by volume is used. Before that, the percentage of solute shown as precipitating out of the system is due to the retention of solution in the membrane similar to the effect observed in the ethanol–acetonitrile case, Section 3.2.1. Any increase in *n*-heptane above 60% in the wash solution shows a significant increase in the amount of solute precipitating out of the solution with around 89% of the dissolved solute precipitating out of the solution when using pure *n*-heptane as the wash solvent. This increase in the amount of precipitation taking place is consistent with the increase in the supersaturation value as the amount of *n*-heptane increases in the system, as seen on the *Y* axis on the right-hand side of graph b.

HPLC of the precipitated crystals was used to determine the composition of the crystals and to quantify the amount of impurities precipitating out of the solution. Graph c in Figure 6 shows the amount of impurities, both metacetamol and acetanilide, that were precipitated in the case of ethanol–*n*-heptane solvent system. There is a gradual increase in the amount of impurity precipitating out of the system after the 0.7 heptane volume fraction at which point precipitation is first detected. Knowing the initial concentration of API and impurities dissolved in the crystallization solution (Table 8), over 10% of the metacetamol and around 5% of the acetanilide impurities were precipitated out of the solution when the wash solution used was pure *n*-heptane.

**Table 8:** Mass of API and Impurities in 120 μL of Ethanolic Solution

**mass of PCM (g)**	0.01769
**mass of metacetamol (g)**	0.00031
**mass of acetanilide (g)**	0.00035

Graph d in Figure 6 shows the ratio of impurity precipitating out compared to the API in the solution, where the ratio is given in eq 1 as
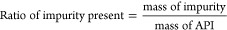
1

As the impurities are uniformly dispersed throughout the solution, the ratios of impurity from 0.1 to 0.6 volume fraction of *n*-heptane in ethanol are relatively constant, graph d, Figure 6. This is because there is no precipitation observed in these samples; the impurities are only present because of the retention of solution in the porous membrane.

After a wash volume fraction of 0.6 *n*-heptane is exceeded, the precipitation of the solute increases; there is a decrease in ratio of impurity precipitating out. Since the amount of impurities in the system is only 2% by mass, at the start of the precipitation process this ratio change is caused by the PCM API that is present in the system precipitating out. When the volume fraction of *n*-heptane in the wash solution reaches 0.8, there is an increase in the ratio of impurity precipitating out with respect to the API. Because the impurity concentrations in the mother liquor are so low, it is unlikely that the impurities are crystallizing as separate crystals. Rather, they are being incorporated in the crystals of PCM. When the volume fraction of *n*-heptane reaches 0.8, around 50% of the PCM solute is precipitated out of the solution and the supersaturation level is around 2; under these conditions, the PCM crystal precipitation is rapid and the impurities are easily incorporated into the API crystals. This affect is also seen in the other two solvent mixture cases where precipitation is observed; isopropanol–*n*-heptane and isoamyl alcohol–*n*-heptane (see the Supporting Information).

**Figure 7:**
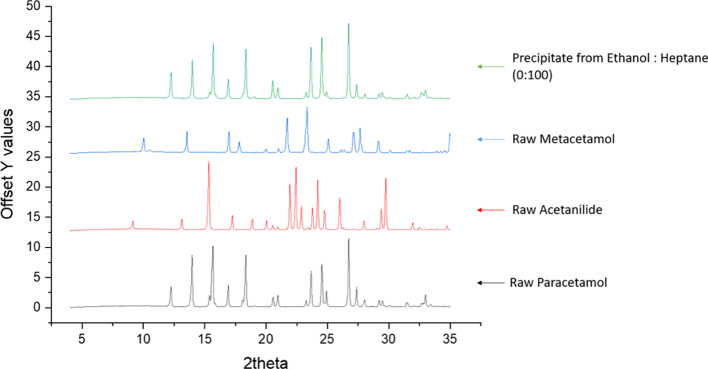
XRPD results for raw PCM, metacetamol, and acetanilide together with the precipitate sample obtained from the ethanol–*n*-heptane sample.

XRPD analysis was performed on the precipitate obtained from the ethanol–*n*-heptane experiments to analyze the structure of the crystalline material. The diffraction data in Figure 7 generated from pure PCM, metacetamol, and acetanilide provide reference XRPDs. From the sample of precipitated material shown in Figure 7, only PCM crystals of form 1 are seen to be present; there are no peaks corresponding to metacetamol or acetanilide. DSC analysis was also performed on the raw materials and the precipitate sample obtained from all three solvent systems where precipitation was detected (Supporting Information). DSC analysis was performed to investigate the effect of the presence of impurities in the precipitate samples. The amounts of impurities in the precipitate samples were found to be smaller than would be needed to be detected because the measured melting temperature of the samples correspond to the melting temperature of pure PCM and no other thermal effect related to the impurity species was observed.

The lack of peaks at 2θ values corresponding to impurities in the XRPD and the absence of significant melting point reduction in the DSC result are presumed to be due to the small amount of impurities present in the precipitate sample compared to the API, as indicated by the HPLC assays. This low concentration of impurities falls below the detection limit of the two techniques, XRPD and DSC, and hence could not be observed.^33,34^

The use of pure *n*-heptane as the wash solvent in the cases examined would not be an ideal washing strategy due to precipitation of both PCM and its impurities of synthesis in the system. Precipitation can be minimized or possibly eliminated by using a two or more-part washing strategy. In the example case, the first wash can be carried out using a 50:50 ethanol/*n*-heptane wash solution. This would allow for most of the saturated ethanoic solution in the API cake to be displaced by the wash solution without causing precipitation. To further improve purity and aid with the drying process, a second wash can then be carried out using pure *n*-heptane to wash out the 50:50 wash solution from the API crystal cake. This washing strategy minimizes the risk of precipitation in the first wash by using a wash solution with a higher solubility limit. Then, a second wash with pure *n*-heptane mitigates the effect of high supersaturation in the system as the pure wash solvent is not coming in contact with the supersaturated mother liquor in the API cake. Also, the residual *n*-heptane in the final deliquored API cake is relatively easily evaporated, and the low solubility of the API, compared to that of the 50:50 ethanol/*n*-heptane wash solution, would ensure quicker drying and also prevent crystalline bridges forming during wash solvent evaporation thereby minimizing agglomeration.

## 4 Conclusions

The quality of the crystalline product which primarily dominated and controlled in the crystallization process is widely influenced by the downstream isolation processes. For optimization of the overall isolation process, it is important to understand and mitigate the adverse effect caused during the washing process. Lack of knowledge and understanding of the washing process can have a dramatic impact on the final crystal product quality achieved at the end of the drying process. Designing an optimum washing regime is crucial to avoid API product batches that are out of specification.

This study investigates wash solvent selection and introduces a simple and material-sparing methodology to help better design washing regimes for API isolation to prevent the risk of impurity precipitation during washing. The glass vial anti-solvent methodology which was developed was found to be very effective as a qualitative evaluation based on the visual detection of precipitation occurring during washing. Effects such as local nucleation can be identified using this method to provide an insight into the kind of process that can be taking place at the washing front inside a saturated API cake during washing.

The centrifuge vial anti-solvent methodology which was developed was found to be very efficient in quantitatively determining the amount of precipitation that can take place during a washing process. The composition of the precipitated crystals can be then determined using the HPLC technique.

In this work, PCM was used as the model compound. The solubility of the API was experimentally determined at different crystallization and wash solvent ratios. The two anti-solvent evaluation methodologies developed in this study are straightforward to conduct and were able to provide a good indication of the effects that would occur within a PCM API cake during washing. The glass vial method readily indicates if precipitation is likely to occur due to the solvent interaction in a washing process. If so, then the centrifuge vial method can be used to determine the extent and composition of the precipitation taking place.

Both of the methods developed are quick and easy to perform and allow for prompt wash solvent evaluation. The qualitative results obtained in the 1 mL glass vial method were successfully replicated in 100 mL volumes. The small sample size required for this technique prevents any solvent wastage and is in line with environmental sustainability. The centrifuge vial method could be further improved by using clear, larger 1 mL vials rather than the opaque 500 μL vials used. However, sourcing such vials with membrane basket compatible with the solvents used in this study proved difficult. Furthermore, using centrifuge vials which would be more solvent-/air-tight would have allowed for mimicking of the glass vial method, where the solvent system could be allowed to equilibrate over 24 h. However, 20 min solvent contact time together with vortex mixing is found to be sufficient, and the three replicates of each experiment obtained similar results with very good repeatability.

From the results, it can be found that the acetonitrile wash solvent did not cause any anti-solvent effect in the case of PCM crystal washing. The use of heptane wash solvent, on the other hand, caused an anti-solvent effect in the case of all three crystallization solvents used in this study. However, these findings alone do not make acetonitrile a good candidate for wash solvent or heptane a poor wash solvent. Developing the right washing strategy and hence choosing the appropriate wash solvent strategy depends on the aim/objective of the washing procedure within the API isolation processes. If the removal of impurity is the main focus, then a wash solvent with high solubility can be used (such as acetonitrile in the case of PCM), but the yield would be adversely affected and there is a significant risk of agglomeration in drying. However, if the complete removal of mother liquor together with minimal effects on the crystal product is the aim, then a multi-step washing strategy should be devised, as exemplified in the ethanol–*n*-heptane solvent mixture example reported in this study. This would allow for the removal of mother liquor with a significantly decreased chance of precipitation occurring and hopefully a corresponding expectation of a reduction in agglomerate formation during drying.

Future work would involve applying the anti-solvent wash selection methodology to other, more complex API products to assist with the wash regime design and scrutinize the versatility of the methodology developed for a wider range of API products.

## Supporting Information Available

Main properties of the solvents used; calculated final ratio of the wash solvent in solution mixtures at the end of wash solution addition; initial experiment conducted of liquid holdup inside the centrifuge vial using water; HPLC calibration curve of PCM; HPLC calibration curve of metacetamol and acetanilide; glass vial showing the presence of liquid solution still present at the bottom of the vial with the solid precipitate; full quantitative analysis of the ethanol–acetonitrile case; quantitative analysis of the ethanol–isopropyl acetate case; quantitative analysis of the isopropanol–acetonitrile case; quantitative analysis of the isopropanol–*n*-heptane case; quantitative analysis of the isopropanol–isopropyl acetate case; quantitative analysis of the isoamyl alcohol–acetonitrile case; quantitative analysis of the isoamyl alcohol–*n*-heptane case; quantitative analysis of the isoamyl alcohol–isopropyl acetate case; XRPD results for raw API (PCM) and it impurities; DSC result obtained for raw API (PCM) and it impurities; and DSC result obtained for the precipitate obtained from the ethanol–heptane system at a 2 °C/min heating rate (PDF)

## Notes

All data underpinning this publication are openly available from the University of Strathclyde KnowledgeBase at https://doi.org/10.15129/caa4490f-be07-4b39-b9db-9e491329c9d6.

## Abbreviations

ICH: International Council for Harmonization of Technical Requirement for Pharmaceuticals for Human use
API: active pharmaceutical ingredient
SEM: scanning electron microscopy
PCM: paracetamol
HPLC: high-pressure liquid chromatography
XRPD: X-ray powder diffraction
DSC: differential scanning calorimetry
Δ*C*: concentration driving force for crystallization

## References

[ref1] Mullin J. W. Crystallizer design and operation. Crystallisation; 4th ed.; Butterworth Heinemann. 2001, Chapter 9.

[ref2] Gao Z.; Rohani S.; Gong J.; Wang J. Recent developments in the crystallisation process: toward the pharmaceutical industry. Engineering 2017, 3, 345–353. 10.1016/j.eng.2017.03.022.

[ref3] McWilliams J. C.; Allian A. D.; Opalka S. M.; May S. A.; Journet M.; Braden T. M. The evolving state of continuing processing in pharmaceutical API manufacturing: a survey of pharmaceutical companies and contract manufacturing organizations. Org. Process Res. Dev. 2018, 22, 1143–1166. 10.1021/acs.oprd.8b00160.

[ref4] Am Ende D. J.. Design of Filtration and Drying Operations. Chemical Engineering in the Pharmaceutical Industry: R&D to Manufacturing; 1st ed.; Wiley: Hoboken. New Jersey, 2011, Chapter 17, pp 315–347.

[ref5] Svarovsky L. Solid-Liquid Separation; 4th ed.; Butterworth-Heinemann: Oxford. U.K., 2000.

[ref6] Guerrero M.; Albet C.; Palomer A.; Guglietta A. Drying of pharmaceutical and biotechnological industries. Food Sci. Technol. Int., 2013, 9, 237–243. 10.1177/1082013203035567.

[ref7] Lim H. L.; Hapgood K. P.; Haig B. Understanding and preventing agglomeration in a filter drying process. Powder Technol. 2016, 300, 146–156. 10.1016/j.powtec.2016.03.003.

[ref8] Tien C. Principles of Filtration; 1st ed.; Elsevier. 2012.

[ref9] Ottoboni S.; Shahid M.; Steven C.; Coleman S.; Meehan E.; Barton A.; Firth P.; Sutherland R.; Price C. J. Developing a batch isolation procedure and running it in an automated semi continuous unit: AWL CFD25 case study. Org. Process Res. Dev. 2020, 24, 520–539. 10.1021/acs.oprd.9b00512. 32336906 PMC7171873

[ref10] Birch M.; Marziano I. Understanding and avoidance of agglomeration during drying processes: a case study. Org. Process Res. Dev. 2013, 17, 1359–1366. 10.1021/op4000972.

[ref11] Capellades G.; Neurohr C.; Azad M.; Brancazio D.; Rapp K.; Hammersmith G.; Myerson A. S. A compact device for integrated filtration, drying, and mechanical processing of active pharmaceutical ingredients. J. Pharm. Sci. 2020, 109, 1365–1372. 10.1016/j.xphs.2019.12.011. 31866299

[ref12] Pakowski Z.; Mujumdar A. S. Drying of Pharmaceutical Product. Handbook of Industrial Drying. 4th ed.; CRC Press, 2007, Chapter 29, pp 689-712.

[ref13] Conder E. W.; Cosbie A. S.; Gaertner J.; Hicks W.; Huggins S.; MacLeod C. S.; Remy B.; Yang B.-S.; Engstrom J. D.; Lamberto D. J.; Papageorgiou C. D. The pharmaceutical drying unit operation: an industry perspective on advancing rhe science and development approach for scale-up and technology transfer. Org. Process Res. Dev. 2017, 21, 420–429. 10.1021/acs.oprd.6b00406.

[ref14] Kuo M. T.; Barrett E. C. Continuous filter cake washing performance. AIChE J. 1970, 16, 633–638. 10.1002/aic.690160421.

[ref15] Ruslim F.; Nirschl H.; Stahl W.; Carvin P. Optimization of the wash liquor flow rate to improve washing of pre-deliquored filter cakes. Chem. Eng. Sci. 2007, 62, 3951–3961. 10.1016/j.ces.2007.04.022.

[ref16] Huhtanen M.; Salmimies R.; Kinnarinen T.; Häkkinen A.; Ekberg B.; Kallas J. Empirical modelling of cake washing in a pressure filter. Sep. Sci. Technol. 2012, 47, 1102–1112. 10.1080/01496395.2011.644877.

[ref17] Specifications: Test Procedures and Acceptance Criteria for New Drug Substances and New Drug Products. International Conference of Harmonisation of Technical Requirements for Registration of Pharmaceuticals for Human Use. ICH Harmonised Tripartite Guideline. : Chemical Substances, 1999. (Q6A).

[ref18] Yazdanpanah N.; Nagy Z. K.; Price C. J.; Barton A.; Coleman S. J. The handbook of continuous crystallisation; RSC, 2020, Chapter 13.

[ref19] Genck W. Make the most of anti-solvent crystallisation. Chem. Process 2010.https://www.chemicalprocessing.com/articles/2010/210/. [cited 2020/05/05]

[ref20] Desiraju G. R.; Vittal J. J.; Ramanan A. Crystal Engineering: A Textbook; World Scientific Publishing, 2011. Chapter 4.

[ref21] Pietsch W. B. The strength of agglomerates bound by salt bridges. Can. J. Chem. Eng. 1969, 47, 403–409. 10.1002/cjce.5450470419.

[ref22] Terdenge L.-M.; Wohlgemuth K. Impact of agglomeration on crystalline product quality within the crystallisation process chain. Cryst. Res. Technol. 2016, 51, 513–523. 10.1002/crat.201600125.

[ref23] Heng J. Y. Y.; Bismarck A.; Lee A. F.; Wilson K.; Williams D. R. Anisotropic surface energetics and wettability of macroscopic form I paracetamol crystals. Langmuir 2006, 22, 2760–2769. 10.1021/la0532407. 16519480

[ref24] Ellis F. Paracetamol–a Curriculum Resource; Royal Society of Chemistry, 2002. ISBN 0-85404-375-6.

[ref25] Prasad K. V. R.; Ristic R. I.; Sheen D. B.; Sherwood J. N. Crystallisation of paracetamol from solution in the presence of impurity. Int. J. Pharm. 2001, 215, 29–44. 10.1016/s0378-5173(00)00653-0. 11250089

[ref26] Hulse W. L.; Grimsey I. M.; De Matas M. The impact of low-level inorganic impurities on key physicochemical properties of paracetamol. Int. J. Pharm. 2008, 349, 61–65. 10.1016/j.ijpharm.2007.07.037. 17884312

[ref27] Di Martino P.; Conflant P.; Drache M.; Huvenne J.-P.; Guyot-Hermann A.-M. Preparation and physical characterization of forms II and III of paracetamol. J. Therm. Anal. 1997, 48, 447–458.

[ref28] Hendriksen B. A.; Grant D. J. W. The effect of structurally related substances on the nucleation kinetics of paracetamol (acetaminophen). J. Cryst. Growth 1995, 156, 252–260. 10.1016/0022-0248(95)00301-0.

[ref29] Kossik J. Small Scale Continuous Cake Filtration using the Disposable Rotary Drum Filter. Filtrat. Separ. 2003, 40, 26–27. 10.1016/S0015-1882(03)00926-1.

[ref30] Granberg R. A.; Rasmuson Å. C. Solublity of Paracetamol in Pure Solvents. J. Chem. Eng. Data 1999, 44, 1391–1395. 10.1021/je990124v.

[ref31] Murugesan S.; Sharma P. K.; Tabora J. E. Design of Filtration and Drying Operations. Chemical Engineering in the Pharmaceutical Industry: R&D to Manufacturing; Wiley: New York, 2010, pp 315–346.

[ref32] Kramer H. J. M.; Van Rosmalen G. M. Crystallisation. Encyclopedia of Seperation Science; Academic Press, 2000. pp 64–84.

[ref33] Dutrow B. L.; Clark C. M. X-ray Powder Diffraction (XRD). Geochemical Instrumentation and Analysis. Carleton College, https://serc.carleton.edu/research_education/geochemsheets/techniques/XRD.html. [cited 2020/05/10]

[ref34] Durowoju I. B.; Bhandal K. S.; Hu J.; Carpick B.; Kirkitadze M. Differential scanning calorimetry–a method for assessing the thermal stability and conformation of protein antigen. J. Visualized Exp. 2017, 4, 55262 10.3791/55262. PMC5409303 28287565

