# Supplementary material for: Exploring the Role of Anti-solvent Effects during Washing on Active Pharmaceutical Ingredient Purity
Source: Org Process Res Dev. 2021 Mar 12;25(4):969–81. doi: 10.1021/acs.oprd.1c00005 (PMC8057229; doi:10.1021/acs.oprd.1c00005)
Supplement: Supplementary file 1 — op1c00005_si_001.pdf [file op1c00005_si_001.pdf]

Supporting Information

Exploring the role of anti-solvent effects during washing on active pharmaceutical ingredient purity

Muhid Shahid[1], Georgia Sanxaridou[1], Sara Ottoboni[1], Leo Lue[2], Chris Price[1,2]*

Table S1: Main properties of the solvents used in this work.[35-39]

**Solvent**	**Boiling point (ºC)**	**Enthalpy of vaporization (kJ/mol)**	**Viscosity (cP) (Temperature ºC)**	**Density (g/ml) (Temperature ºC)**	**Surface tension (mN/m) (Temperature ºC)**
Ethanol	78.4	38.58	1.26 (20)	0.79 (20)	21.99 (20)
Isopropanol	82.2	39.85	2.1 (25)	0.78 (25)	21.4 (20)
Isoamyl alcohol	132	55.2	3.74 (25)	0.81 (15)	24.77 (15)
Acetonitrile	81.6	33.23	0.35 (20)	0.78 (20)	29.04 (20)_
n-Heptane	98.4	31.77	0.397 (25)	0.68 (20)	19.7 (20)
Isopropyl acetate	88.5	37.2	0.52 (25)	0.87 (20)	22.3 (20)

Table S2: Calculated final ratio of wash solvent in solution mixtures at the end of wash solution addition.

**Starting volume of crystallisation solvent (µl)**	300						
**Ratio of wash solution (crystallisation : wash)**	90:10	75:25	50:50	40:60	30:70	20:80	10:90	0:100
Volume of crystallisation solvent in wash solution (µl)	630	525	350	280	210	140	70	0
Volume of wash solvent in wash solution (µl)	70	175	350	420	490	560	630	700
Final volume of crystallisation solvent in solution (µl)	930	825	650	580	510	440	370	300
Final volume of wash solvent in solution (µl)	70	175	350	420	490	560	630	700
**Therefore volume fraction of wash solvent in final solution**	0.07	0.175	0.35	0.42	0.49	0.56	0.63	0.7

Table S3: Initial experiment conducted of liquid holdup inside centrifuge vial using water. The rpm was set at 6000. The amount of time of centrifugation was varied. The cells with italic and bold numbering are the one where the vials were left in the centrifuge for an extra 2-3 minutes before taking the sample out and measuring the mass. This extra time helped in draining much more of the solvent out of the filter and so resulted in much less solvent hold-up in those samples.

Vial	Weight of centrifuge filter tare (g)	Weight of centrifuge filter after 1 min centrifuging (g)	Mass of solvent holdup in the filter after 1 minute (g)	Weight of centrifuge filter after another 1 min centrifuging (g)	Mass of solvent holdup in the filter after 2 minute (g)
1	0.40129	0.42540	0.02411	0.41587	0.01458
2	0.40213	0.41018	***0.00805***	0.40592	***0.00379***
3	0.40235	0.40870	***0.00635***	0.40563	***0.00328***
4	0.40125	0.43443	0.03318	0.4052	0.00395
		**Average holdup**	**0.01792**		**0.0064**

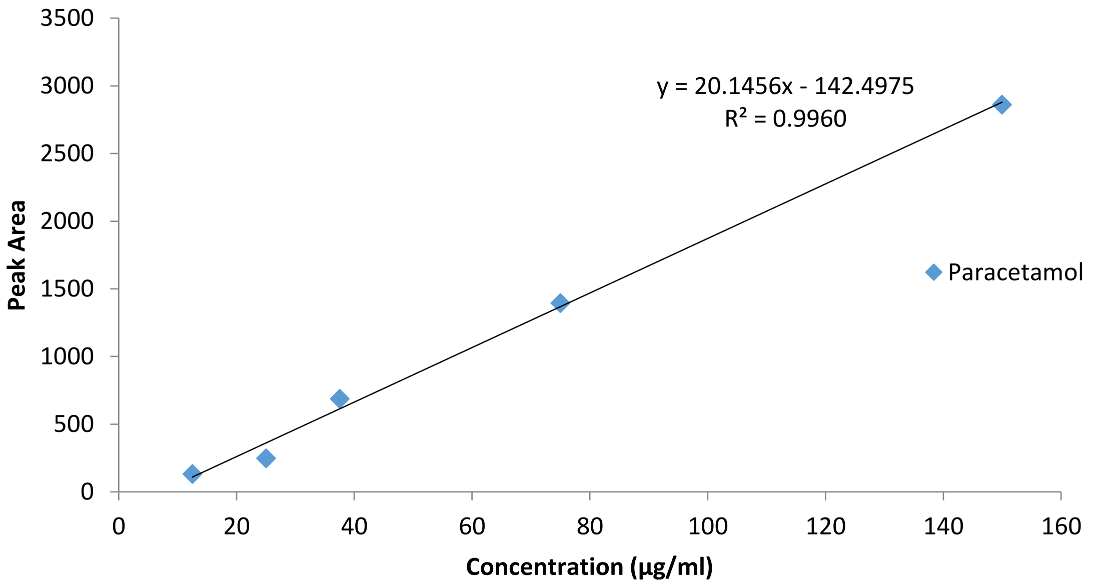

Figure S1: HPLC calibration curve of paracetamol.

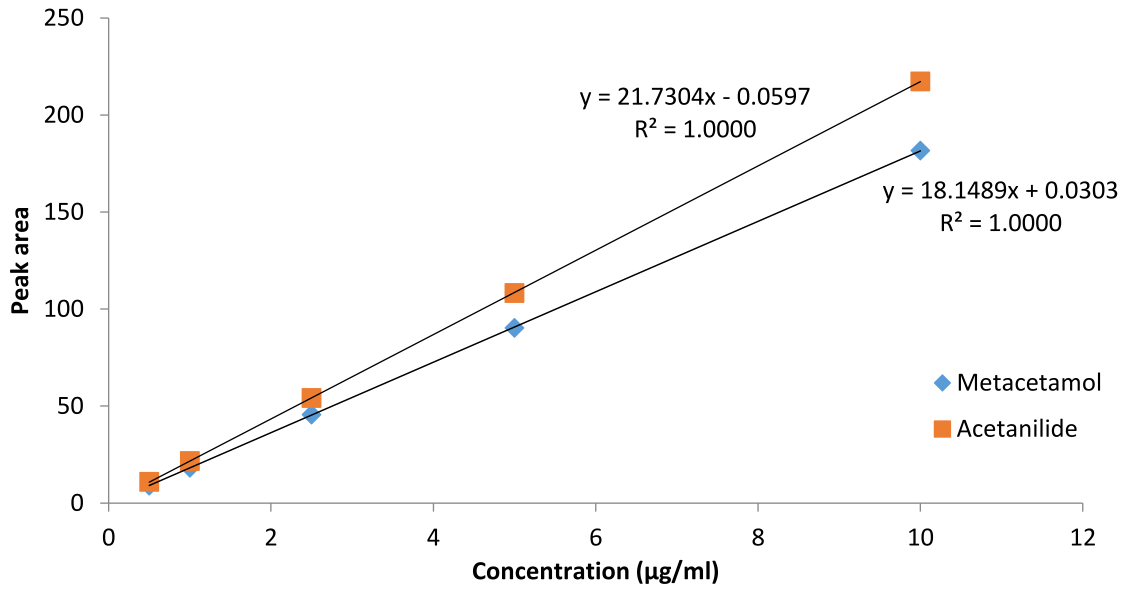

Figure S2: HPLC calibration curve of metacetamol and acetanilide.

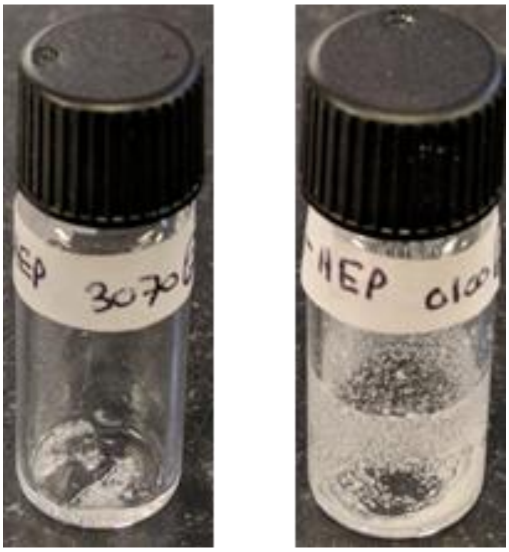

Figure S3: Glass vial showing presence of liquid solution still present at the bottom of the vial with the solid precipitate (incomplete separation of solid and liquid sample).

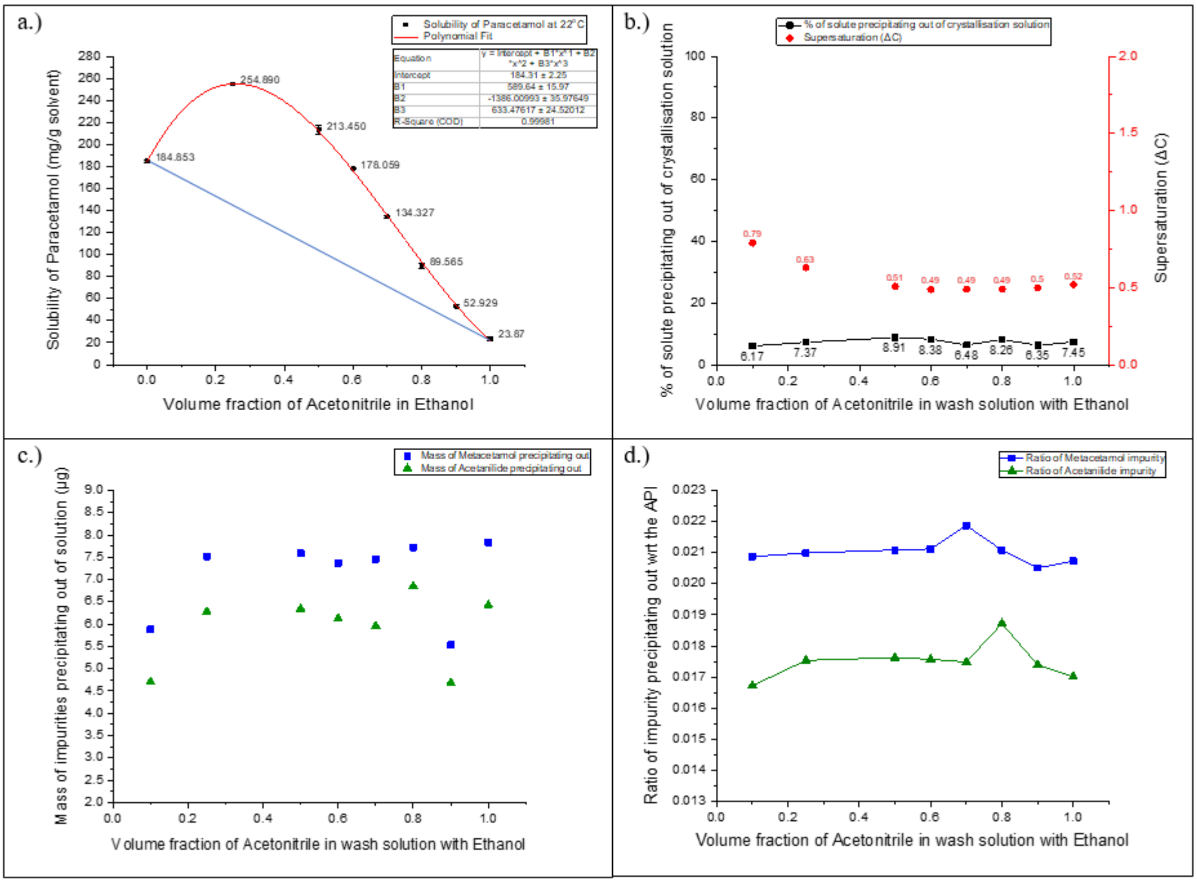

Figure S4: Full quantitative analysis of ethanol-acetonitrile case. a.) Solubility of paracetamol in ethanol-acetonitrile binary solvent mixture at 22 °C. b.) Percentage of solute precipitating out of solution for different wash solution is shown in the graph together with the supersaturation achieved in the solution when different ratio of wash solution is added to the saturated crystallisation solvent. c.) Mass of impurities precipitating out when using different ratios of wash solution. d.) Ratio of impurities precipitating out with respect to the paracetamol (API) for each of the different ratios of wash solutions used.

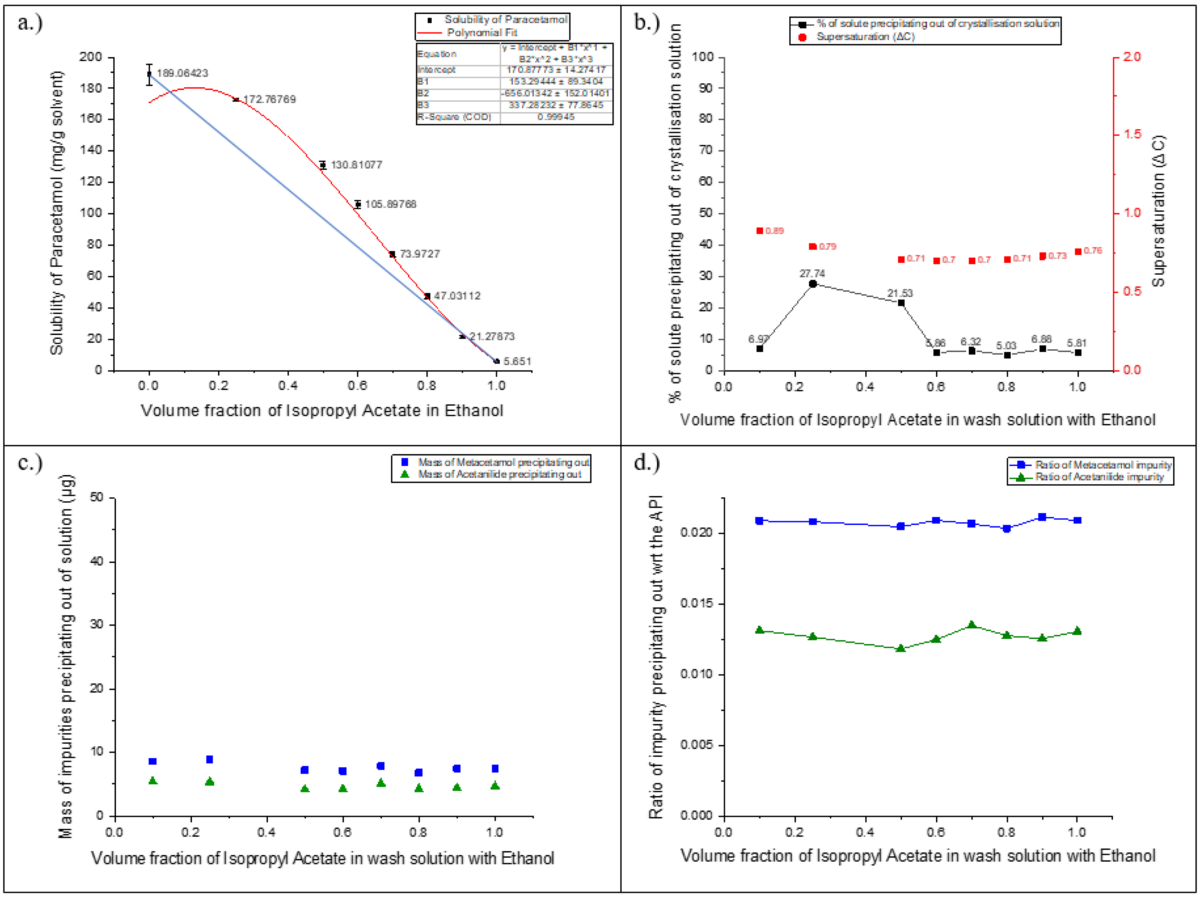

Figure S5: Quantitative analysis of ethanol-isopropyl acetate case. a.) Solubility of paracetamol in ethanol-isopropyl acetate binary solvent mixture at 22 °C. b.) Percentage of solute precipitating out of solution for different wash solution is shown in the graph together with the supersaturation achieved in the solution when different ratio of wash solution is added to the saturated crystallisation solvent. c.) Mass of impurities precipitating out when using different ratios of wash solution. d.) Ratio of impurities precipitating out with respect to the paracetamol (API) for each of the different ratios of wash solutions used.

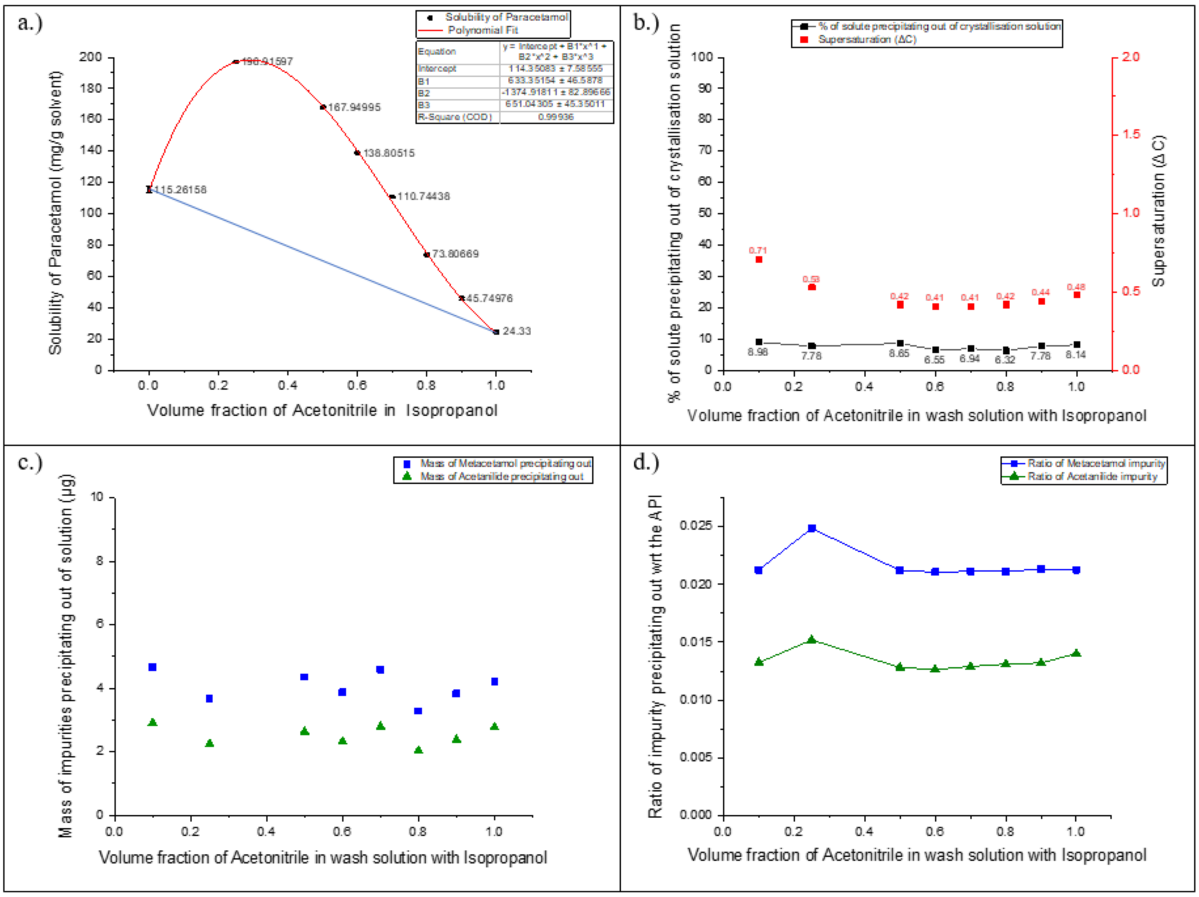

Figure S6: Quantitative analysis of isopropanol-acetonitrile case. a.) Solubility of paracetamol in isopropanol-acetonitrile binary solvent mixture at 22 °C. b.) Percentage of solute precipitating out of solution for different wash solution is shown in the graph together with the supersaturation achieved in the solution when different ratio of wash solution is added to the saturated crystallisation solvent. c.) Mass of impurities precipitating out when using different ratios of wash solution. d.) Ratio of impurities precipitating out with respect to the paracetamol (API) for each of the different ratios of wash solutions used.

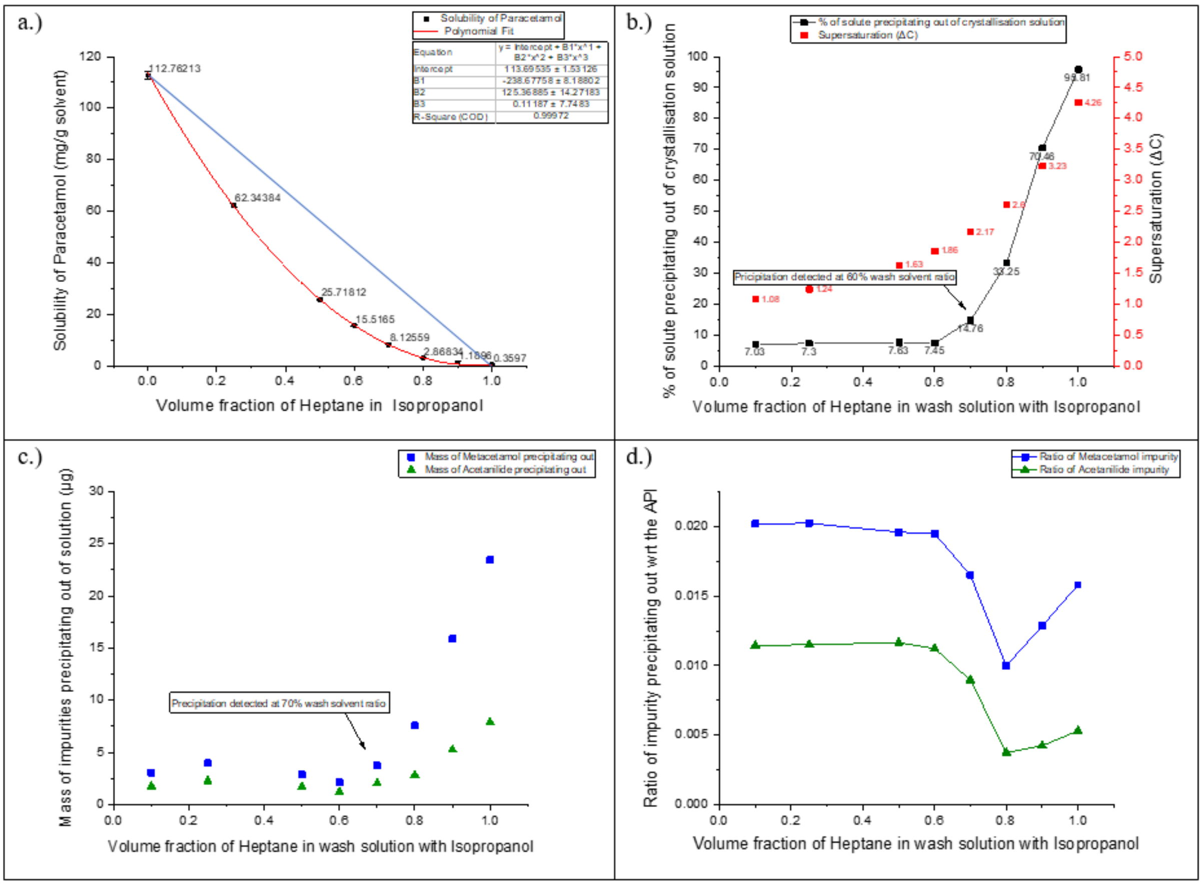

Figure S7: Quantitative analysis of isopropanol-heptane case. a.) Solubility of paracetamol in isoprpanol-heptane binary solvent mixture at 22 °C. b.) Percentage of solute precipitating out of solution for different wash solution is shown in the graph together with the supersaturation achieved in the solution when different ratio of wash solution is added to the saturated crystallisation solvent. c.) Mass of impurities precipitating out when using different ratios of wash solution. d.) Ratio of impurities precipitating out with respect to the paracetamol (API) for each of the different ratios of wash solutions used.

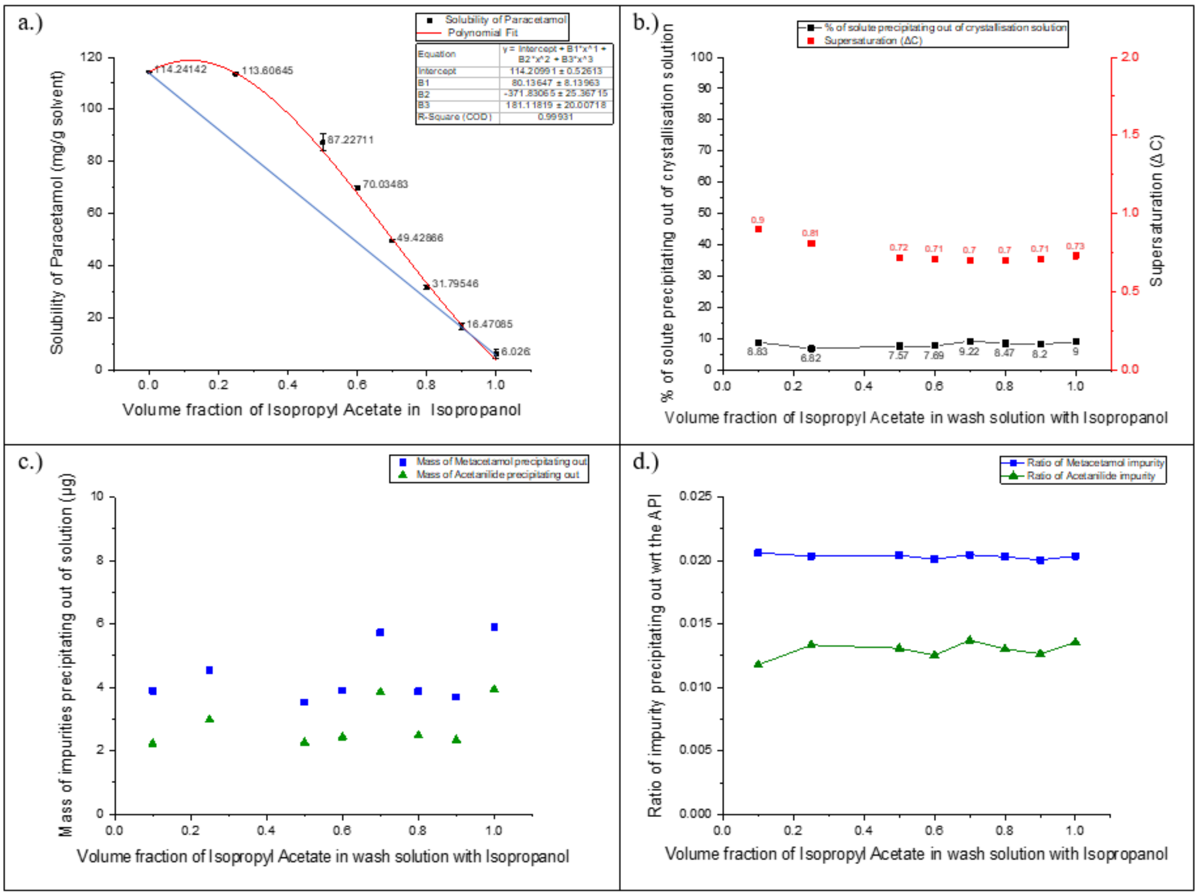

Figure S8: Quantitative analysis of isopropanol-isopropyl acetate case. a.) Solubility of paracetamol in isopropanol-isopropyl acetate binary solvent mixture at 22 °C. b.) Percentage of solute precipitating out of solution for different wash solution is shown in the graph together with the supersaturation achieved in the solution when different ratio of wash solution is added to the saturated crystallisation solvent. c.) Mass of impurities precipitating out when using different ratios of wash solution. d.) Ratio of impurities precipitating out with respect to the paracetamol (API) for each of the different ratios of wash solutions used.

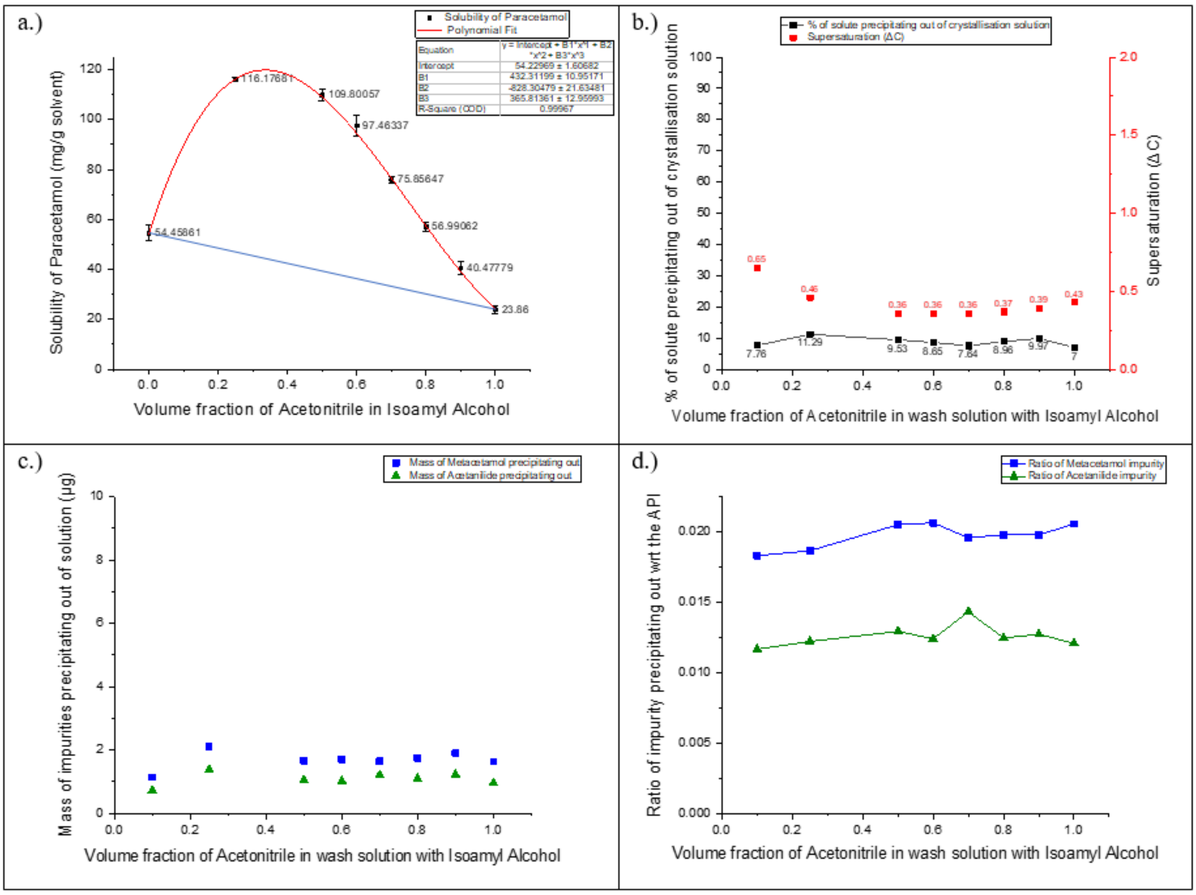

Figure S9: Quantitative analysis of isoamyl alcohol-acetonitrile case. a.) Solubility of paracetamol in isoamyl alcohol-acetonitrile binary solvent mixture at 22 °C. b.) Percentage of solute precipitating out of solution for different wash solution is shown in the graph together with the supersaturation achieved in the solution when different ratio of wash solution is added to the saturated crystallisation solvent. c.) Mass of impurities precipitating out when using different ratios of wash solution. d.) Ratio of impurities precipitating out with respect to the paracetamol (API) for each of the different ratios of wash solutions used.

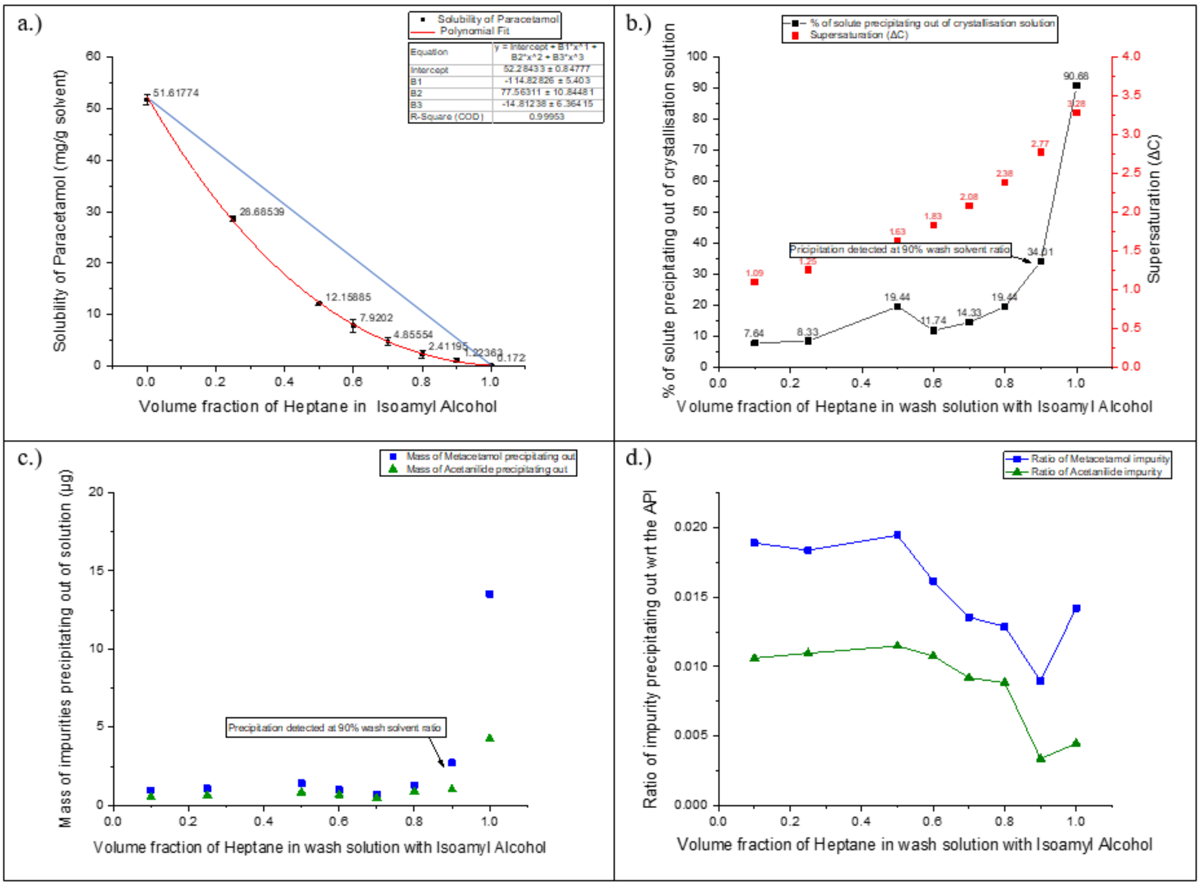

Figure S10: Quantitative analysis of isoamyl alcohol-heptane case. a.) Solubility of paracetamol in isoamyl alcohol-heptane binary solvent mixture at 22 °C. b.) Percentage of solute precipitating out of solution for different wash solution is shown in the graph together with the supersaturation achieved in the solution when different ratio of wash solution is added to the saturated crystallisation solvent. c.) Mass of impurities precipitating out when using different ratios of wash solution. d.) Ratio of impurities precipitating out with respect to the paracetamol (API) for each of the different ratios of wash solutions used.

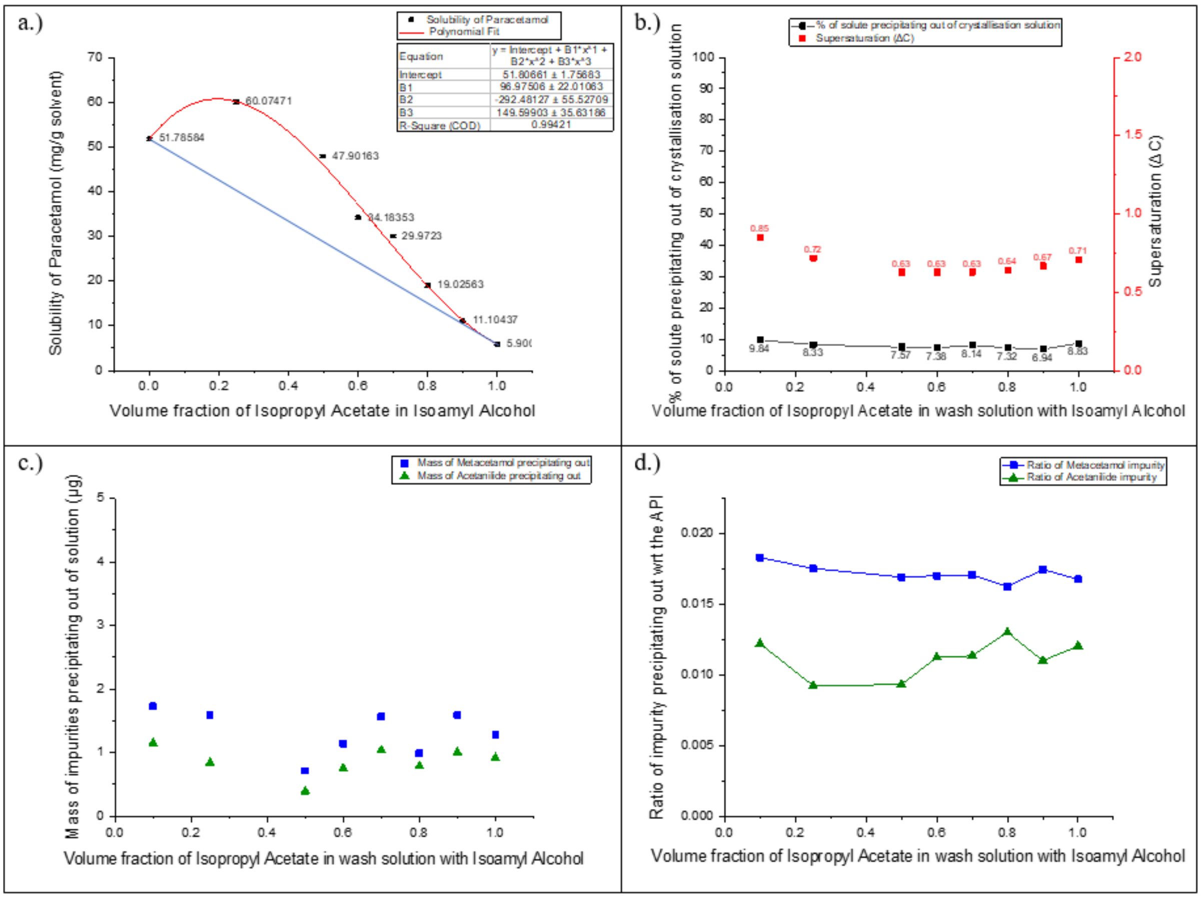

Figure S11: Quantitative analysis of isoamyl alcohol-isopropyl acetate case. a.) Solubility of paracetamol in isoamyl alcohol-isopropyl acetate binary solvent mixture at 22 °C. b.) Percentage of solute precipitating out of solution for different wash solution is shown in the graph together with the supersaturation achieved in the solution when different ratio of wash solution is added to the saturated crystallisation solvent. c.) Mass of impurities precipitating out when using different ratios of wash solution. d.) Ratio of impurities precipitating out with respect to the paracetamol (API) for each of the different ratios of wash solutions used.

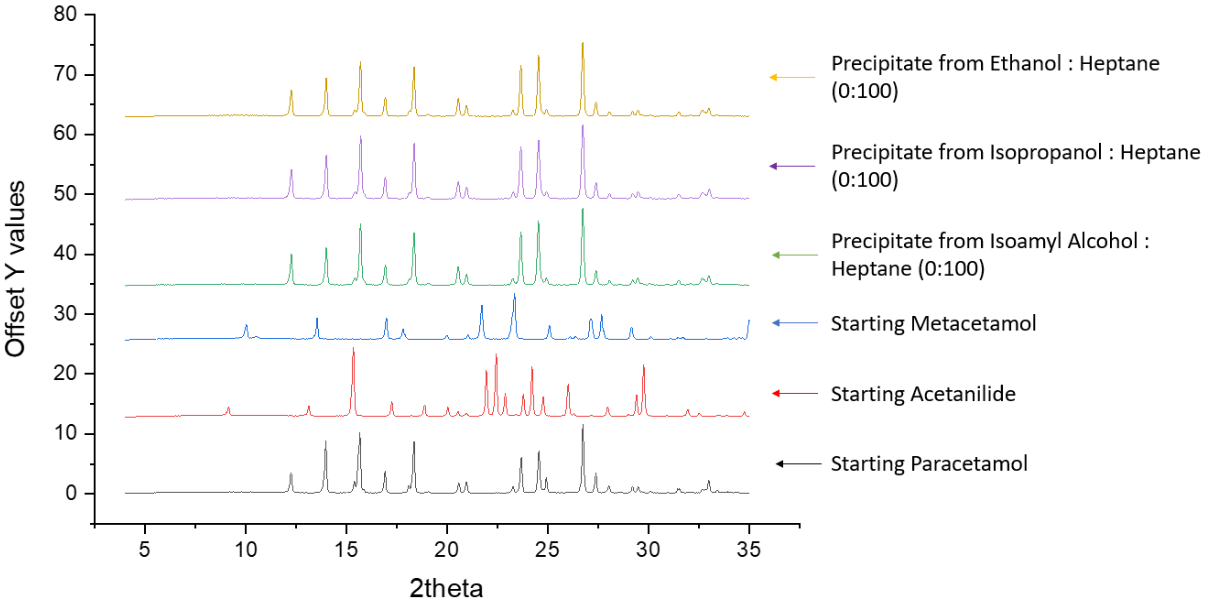

Figure S12: XRPD results for raw API (paracetamol) and it impurities (metacetamol and acetanilide) together with the precipitate obtained from some sample wash solution results.

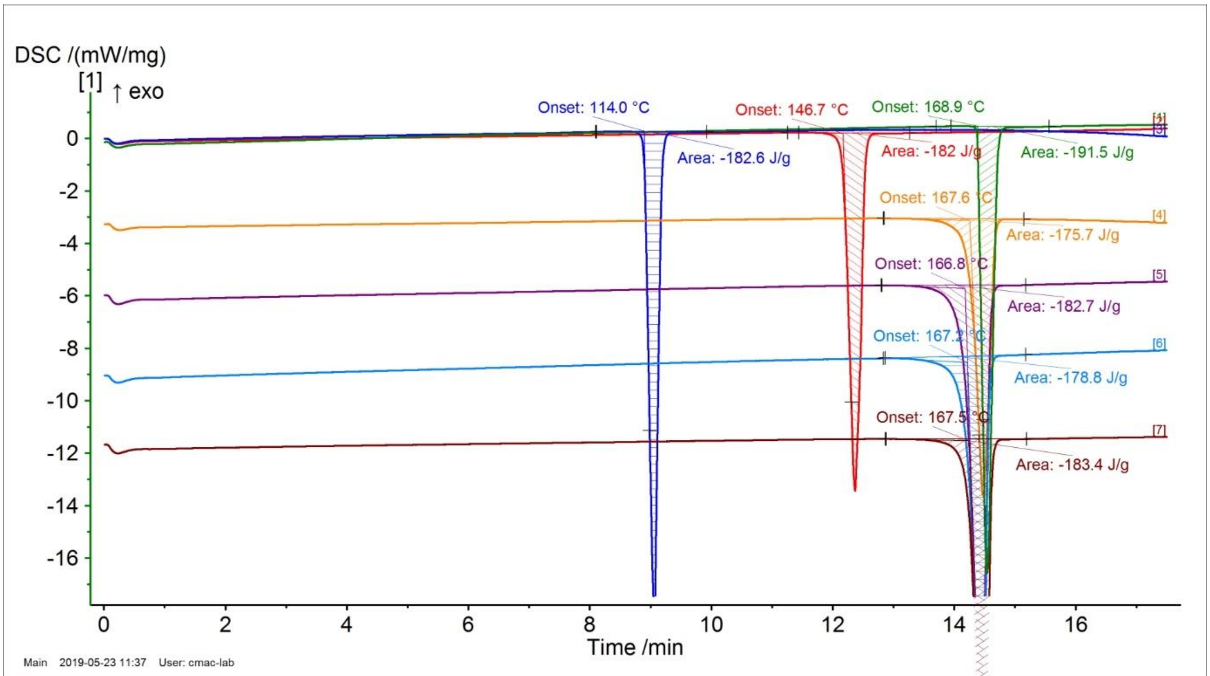

Main 2019-05-23 11:37 User: cmac-lab

[#] Instrument	File	Date	Identity	Sample	Mass/mg	Segment	Range	Atmosphere	Corr.
[1] DSC 214 Polyma	strtPCM.ngb-ddg	2019-05-22	strtPCM	StrtPCM	2.33	1/1	25°C/10.0(K/min)/200°C	He, 40.0ml/min / He, 60.0ml/min	020
[2] DSC 214 Polyma	strtMeta.ngb-ddg	2019-05-22	strtMeta	srtrMeta	3.14	1/1	25°C/10.0(K/min)/200°C	He, 40.0ml/min / He, 60.0ml/min	020
[3] DSC 214 Polyma	strtAceta.ngb-ddg	2019-05-22	strtAceta	strtAceta	2.12	1/1	25°C/10.0(K/min)/200°C	He, 40.0ml/min / He, 60.0ml/min	020
[4] DSC 214 Polyma	EthHep0100.ngb-ddg	2019-05-22	EthHep0100	EthHep0100	2.93	1/1	25°C/10.0(K/min)/200°C	He, 40.0ml/min / He, 60.0ml/min	020
[5] DSC 214 Polyma	IsmHep0100.ngb-ddg	2019-05-22	IsmHep0100	IsmHep0100	3.11	1/1	25°C/10.0(K/min)/200°C	He, 40.0ml/min / He, 60.0ml/min	020
[6] DSC 214 Polyma	Isphep0100.ngb-ddg	2019-05-22	Isphep0100	Isphep0100	2.68	1/1	25°C/10.0(K/min)/200°C	He, 40.0ml/min / He, 60.0ml/min	020
[7] DSC 214 Polyma	IspHep2080.ngb-ddg	2019-05-22	IspHep2080	IspHep2080	3.24	1/1	25°C/10.0(K/min)/200°C	He, 40.0ml/min / He, 60.0ml/min	020

Created with NETZSCH Proteus software

Figure S13: DSC result obtained for raw API (paracetamol) and it impurities (metacetamol and acetanilide) together with the precipitate obtained from some sample wash solution results at 10 ^{o}C/min heating rate.

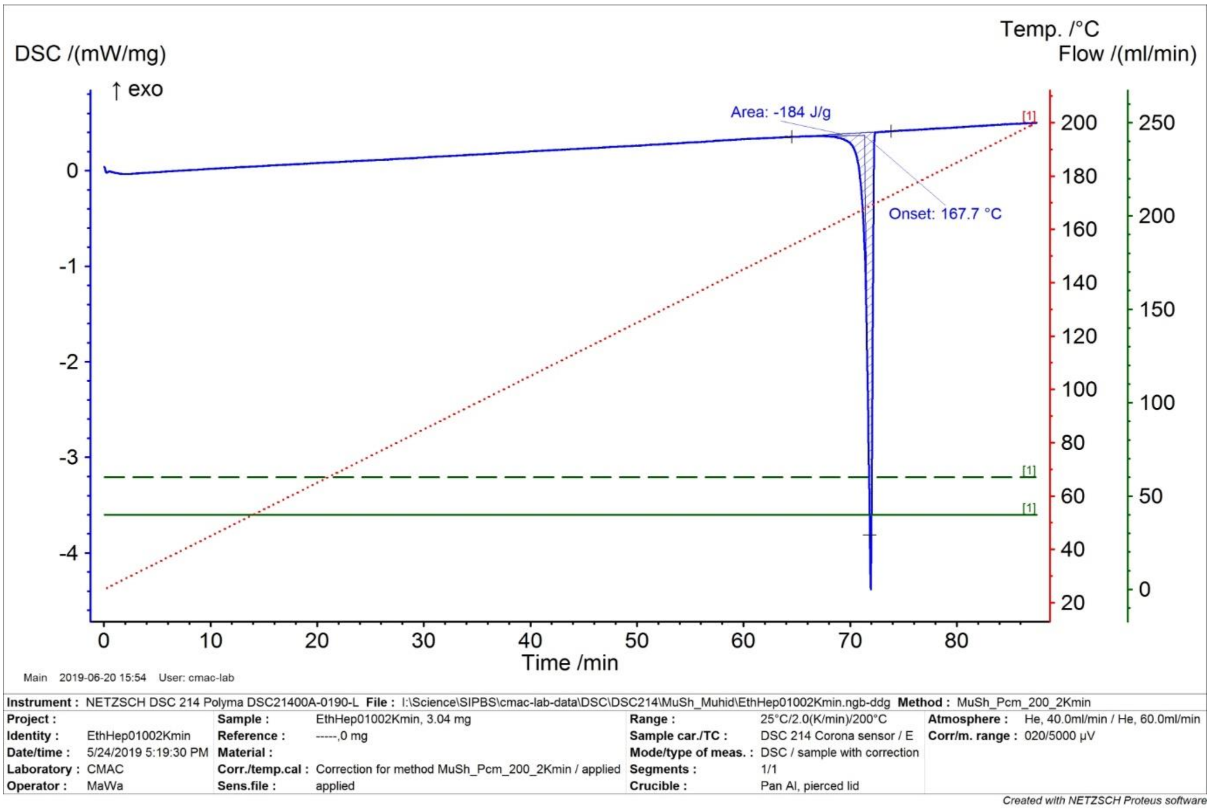

Figure S14: DSC result obtained for precipitate obtained from ethanol-heptane system at 2 ^{o}C/min heating rate.
